# CXCR4 has a dual role in improving the efficacy of BCMA-redirected CAR-NK cells in multiple myeloma

**DOI:** 10.3389/fimmu.2024.1383136

**Published:** 2024-06-24

**Authors:** Michael W. Moles, Henry Erdlei, Lutz Menzel, Marialucia Massaro, Agnese Fiori, Mario Bunse, Moritz Schrimpf, Kerstin Gerlach, Venugopal Gudipati, John Reiser, Ketan Mathavan, Jodie P. Goodrich, Johannes B. Huppa, Jan Krönke, Bahram Valamehr, Uta E. Höpken, Armin Rehm

**Affiliations:** ^1^ Translational Tumorimmunology, Max Delbrück Center, Berlin, Germany; ^2^ Microenvironmental Regulation in Autoimmunity and Cancer, Max Delbrück Center, Berlin, Germany; ^3^ Center for Pathophysiology, Infectiology and Immunology, Institute for Hygiene and Applied Immunology, Medical University of Vienna, Vienna, Austria; ^4^ Fate Therapeutics, San Diego, CA, United States; ^5^ Department of Hematology, Oncology and Tumorimmunology, Charité-University Medicine Berlin, Berlin, Germany

**Keywords:** BCMA, chimeric antigen receptor, NK cells, multiple myeloma, chemokine receptor CXCR4, adoptive T cell therapy

## Abstract

Multiple myeloma (MM) is a plasma cell disease with a preferential bone marrow (BM) tropism. Enforced expression of tissue-specific chemokine receptors has been shown to successfully guide adoptively-transferred CAR NK cells towards the malignant milieu in solid cancers, but also to BM-resident AML and MM. For redirection towards BM-associated chemokine CXCL12, we armored BCMA CAR-NK-92 as well as primary NK cells with ectopic expression of either wildtype CXCR4 or a gain-of-function mutant CXCR4^R334X^. Our data showed that BCMA CAR-NK-92 and -primary NK cells equipped with CXCR4 gained an improved ability to migrate towards CXCL12 *in vitro*. Beyond its classical role coordinating chemotaxis, CXCR4 has been shown to participate in T cell co-stimulation, which prompted us to examine the functionality of CXCR4-cotransduced BCMA-CAR NK cells. Ectopic CXCR4 expression enhanced the cytotoxic capacity of BCMA CAR-NK cells, as evidenced by the ability to eliminate BCMA-expressing target cell lines and primary MM cells *in vitro* and through accelerated cytolytic granule release. We show that CXCR4 co-modification prolonged BCMA CAR surface deposition, augmented ZAP-70 recruitment following CAR-engagement, and accelerated distal signal transduction kinetics. BCMA CAR sensitivity towards antigen was enhanced by virtue of an enhanced ZAP-70 recruitment to the immunological synapse, revealing an increased propensity of CARs to become triggered upon CXCR4 overexpression. Unexpectedly, co-stimulation via CXCR4 occurred in the absence of CXCL12 ligand-stimulation. Collectively, our findings imply that co-modification of CAR-NK cells with tissue-relevant chemokine receptors affect adoptive NK cell therapy beyond improved trafficking and retention within tumor sites.

## Introduction

Chimeric antigen receptor (CAR)-T cell immunotherapy has emerged as an effective therapeutic option for relapsed and refractory multiple myeloma (MM) (1, 2). Results from the pivotal KarMMa (3) and CARTITUDE-1 (4, 5), studies have demonstrated the efficaciousness of BCMA CAR-T cell therapy to induce deep remissions for heavily pretreated MM patients. Nevertheless, the majority of recipients eventually relapse (3, 4, 6).

Mechanisms of acquired resistance to BCMA CAR-T cell therapy include immune escape due to target antigen loss. The exact mechanism for immune escape is more complex than previously reported for CD19 CAR-T cells since complete BCMA antigen loss is less frequent (7, 8). Other CAR-T cell- and microenvironmental-related factors for treatment failure include CAR-T cell exhaustion, limited migration to and persistence within the tumor niche, and the immunosuppressive BM microenvironment. Furthermore, the generation of suboptimal autologous CAR-T cells due to T cell dysfunction in pre-treated patients (9) has prompted the development of ‘next generation’ cellular products to improve patient outcomes (10).

CAR-modified natural killer (NK) cells are a powerful, allogeneic alternative to autologous CAR-T cells. While exhibiting comparable effector functions, off-the-shelf CAR-NK cells offer several advantages over CAR-T cells including an intrinsic ability to eliminate tumor cells in an HLA-unrestricted manner, a favorable safety profile with a negligible risk of graft-versus-host disease, or cytokine release syndrome, and more economical methods for CAR-NK production (11). Indeed, clinical studies demonstrated the efficacy and safety of CAR-NK cells derived from cord blood (12), induced-pluripotent stem cells (iPSCs) (13) and cell lines (14). Nevertheless, effective anti-tumor immunity is reliant upon the ability of adoptively transferred NK cells to breach the tumor niche (15). NK cells navigate towards malignant tissues *via* their chemokine receptors, which respond to cognate chemokines expressed in the tumor microenvironment (TME) (11). E*x vivo*-manipulation of primary NK cells has been shown to hinder penetration of the TME, in part, due to chemokine receptor downmodulation (16–18). For these reasons, engineering strategies to stably equip effector NK cells with tissue-relevant chemokine receptors have shown promise in pre-clinical studies for solid tumors (18–21) and hematological malignancies (16, 22, 23).

NK cell cytotoxic function is exerted without prior immune sensitization through secretion of perforin and granzyme-containing lytic granules. A prerequisite is the formation of the immunological synapse (IS) (24), and after target cell conjugation, granule content is released within minutes and can effectuate pro-apoptotic pathways in target cells in a serial manner (25). NK cells can also kill tumor cells by caspase-dependent death receptor engagement by their cognate ligands, e.g., TRAIL and FasL (26). These effector functions are regulated by several activating and inhibitory receptors which simultaneously recognize a multitude of ligands expressed on target cells. A dominance of NK cell activation receptor signals endows NK cells with innate abilities to mediate effector functions, i.e., NKG2D signaling is mediated through the adaptor molecules DAP10 and DAP12, where DAP12 recruits ZAP-70 and Syk to initiate NK cell activation (27–29). Receptor-proximal signaling molecules activate the signaling cascade, containing AKT, MAPKs, ERK1/2, JNK1/2, and p38. Alternatively, NK cells can couple *via* CD16/FcγRIIIA to target cells decorated with a therapeutic antibody (30).

The homeostatic chemokine receptor CXCR4 coordinates trafficking of NK cells towards the BM niche, where its ligand CXCL12 is constitutively expressed. Problematically for adoptive CAR-NK cell therapy of MM, CXCR4 expression is progressively lost during NK cell maturation, coinciding with an increase of the chemokine receptor CXCR3, which generally promotes NK cell mobilization from the BM into the periphery (31). NK cell homing to and retention within the BM has been shown to correlate with improved acute myeloid leukemia (AML) control (15, 32) However, the chemokine TME is sufficiently skewed during MM development, whereby CXCL12 downmodulation is paralleled by increased expression of CXCR3 cognate ligands, creating pathological conditions generally restrictive to NK cell infiltration (22). This provides the rationale for reestablishing the responsiveness of NK cells to BM-associated CXCL12 to improve MM disease control. Beyond their classical role coordinating chemotaxis, previous reports in T cells have shown that recruitment of chemokine receptors within the IS serves to enhance T cell activation (33–35). To this date, a role for chemokine receptors as co-receptors in CAR-NK cell activation has not been described.

Here we asked to what degree does CXCR4 impact BCMA CAR-NK cell anti-tumor efficacy beyond BM homing and accumulation. We explored whether enforced CXCR4 expression in NK cells exerted a co-stimulatory role in the context of CAR-induced NK cell activation. Based on our findings we conclude that co-modification of BCMA CAR-NK cells with CXCR4 exceeds functionalities related to improved trafficking and retention within tumor sites.

## Materials and methods

### Generation of γ-retroviral vectors

The fully humanized anti-human BCMA CAR was generated and used as described (36). The human CXCR4 coding sequence was cloned downstream to the anti-human BCMA CAR sequence linked via a self-cleaving peptide P2A. The entire CAR and CXCR4 sequences were synthesized by GeneArt, and cloned into the retroviral vector MP71. For construction of a CXCR4-only expressing vector, a PCR reaction with primers to introduce a NotI and a EcoRI restriction site was run on the human CXCR4 coding sequence downstream of the aforementioned P2A site contained in a MP71 retroviral vector. The MP71 plasmid was digested with NotI and EcoRI, followed by ligation of the CXCR4 PCR sequence into the vector backbone.

The mutant form CXCR4^R334X^ was generated by Gibson assembly. Briefly, the MP71 plasmid BCMA CAR-CXCR4 was digested with BsteII and EcoRI. An overhang extension PCR was used to create a CXCR4-fragment with a stop codon at amino acid position 334. For construction of all other CXCR4 mutant forms, CXCR4 was cut with BsteII and EcoRI and replaced with mutant CXCR4-fragment R134N (CXCR4^G protein-def.^) sequences. All sequences were verified by restriction digest and Sanger sequencing.

### Cytotoxicity assay

NK-92 and YTS NK cell lines cells stably transduced with various CAR constructs were seeded in 96-well U-bottom plates and mixed with a fixed number of 5x10^4^ Raji^BCMA^ target cells. SP6-CAR NK-92 cells were used as a negative control, and tumor cells only were used for calculation of NK-92 cell-specific killing. Co-cultures were kept for 17 hours and stopped by inclusion of ice-cold FACS buffer. Cells were resuspended in blocking buffer supplemented with Fx True stain. To distinguish effector and target cells, anti-CD56-PE-Cy7 and anti-CD19-FITC antibodies in combination with 7-AAD for dead cell exclusion were used. Samples were acquired on a FACS Canto II flow cytometer (BD Biosciences), or a MACSQuant X analyzer (Miltenyi Biotec) and further analyzed with FlowJo v. 10.0.8 software.

### Multiple myeloma cell line and primary multiple myeloma cells

Bone marrow-derived cells from three MM patients were obtained freshly and further purified using a Pancoll gradient before freezing. The study was conducted according to the Declaration of Helsinki and in accordance with local ethical guidelines; written informed consent of all patients was obtained. The diagnosis of MM was made by expert Charité-University Medicine Berlin hematologists and pathologists, and it integrates cell morphology, immunohistology, marker expression in flow cytometry, and pathophysiological behavior. Thawed target cells were used in co-cultures similar to MM.1S-GFP^+^ cell line. NK-92 effector cells in co-culture were labeled with eFluor670 or with anti-CD56, and after 6 hours target MM cells were defined by CD38^high^ and eFluor670**^-^**. A 7-AAD staining was used for live-dead cell discrimination.

### BCMA-bead stimulation and signal transduction

1x10^6^ CAR-transduced NK-92 cells were pelleted by centrifugation at 400x g for 5 min. Cells were resuspended with BCMA protein-coupled magnetic beads (6 μg/mL; ACRO Biosystems) in PBS. A biotinylated BCMA fragment covers the N-terminal aa 1–54 that includes the cognate epitope for the scFv BCMA. BCMA-beads were added to NK-92 cells at 37°C for 5 min. Following the incubation period, the cells were placed on ice and lysed with ice-cold 2X RIPA buffer (300 mM NaCl, 100 mM Tris-HCl, 2% NP-40, 1% C_24_H_39_NaO_4_, 0.2% SDS) containing protease and phosphatase inhibitors. The lysate was incubated on ice for 30 min and then centrifuged at 13,000 g for 20 min. Supernatant was processed for immunoblot analysis.

### Endocytosis assay

To quantitate the kinetics of BCMA-CAR surface downregulation, CAR-transduced NK-92 cells were seeded in 1 ml of RPMI/0.5% BSA medium and kept at 37°C in a waterbath. 100 μl BCMA protein-coupled magnetic beads (Acro Biosystems) were washed, resuspended in medium and added to the cell suspension at 37°C. Cells were incubated for 10 min, centrifuged and resuspended in medium at 37°C. Aliquots were taken and added to an equal volume of ice-cold FACS buffer (PBS, 0.5% BSA, 0.05% NaN_3_). Cell suspensions were immediately centrifuged at 4°C and resuspended in FACS buffer. For detection of CARs, a PE-coupled goat anti-human IgG antibody (Southern Biotech) was used. Cells were then centrifuged and resuspended in fixation buffer (BioLegend), followed by analysis on a FACS Canto II instrument.

### Ethics approval

The recruitment of voluntary blood and bone marrow donors was conducted according to the declaration of Helsinki and in accordance with local ethical guidelines. The study was approved by the responsible ethic committee at Charité-University Medicine Berlin (EA2/142/20).

## Results

### CAR-CXCR4 co-expression improves migration of NK cells towards BM-associated CXCL12 *in vitro*

To gauge the migration of NK cells towards tissue-specific chemoattractants, we measured expression levels of chemokine receptors CXCR3, CXCR4 and CCR6 and adhesion molecule CD62L on the clinically relevant cell line NK-92. NK-92 cells were devoid of CCR6 and CD62L, possessed negligible levels of CXCR4 and ubiquitously expressed high levels of CXCR3 (**Supplementary Figures S1A, B**).

**Figure 1:**
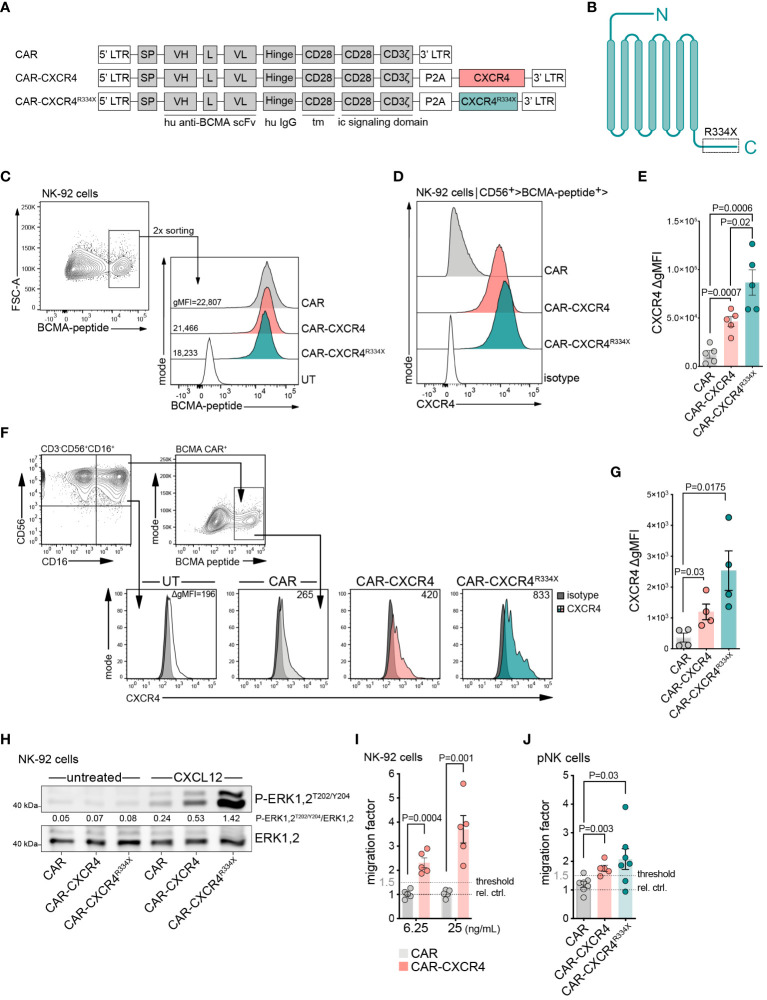
Enforced CXCR4 expression improves migration of BCMA-CAR NK cells towards chemotactic CXCL12 gradients. **(A)** Schematic of retroviral constructs encoding a BCMA-CAR (CAR), equipped with either a WT CXCR4 (CAR-CXCR4) or mutant CXCR4 (CAR-CXCR4^R334X^) chemokine receptor sequence. LTR, long terminal repeat; tm, transmembrane region; ic, intracellular signaling domain; SP, signal peptide. **(B)** Schematic of heptahelical CXCR4^R334X^ mutant. **(C)** FACS plots depicting the generation of BCMA-CAR^+^ NK-92 cells. NK-92 cells underwent retroviral transduction with the aforementioned BCMA-CAR-encoding constructs. BCMA-CAR^+^ expressing NK-92 cells, as detected by cognate FITC-conjugated BCMA-peptide, were enriched twice by FACS to generate stable BCMA-CAR^+^ NK-92 cell lines. **(D)** Of these BCMA-CAR^+^ NK-92 cells, the expression of CXCR4 was assessed by flow cytometry, which was **(E)** quantified as ΔgMFI (values represent CXCR4 stained cells minus isotype control). Statistics calculated by an unpaired t-test, error bars ± SEM. **(F)** FACS histograms showing CXCR4 expression on pNK cells at day +9 of *ex vivo* expansion (2-days post-transduction; untransduced (UT); isotype, dark filled histogram; one representative example is shown. The gating strategy used to define CD56^+^CD16^+^ pNK cells is shown on the left plot. NK cells were stimulated with IL-2 (500 U/ml) and IL-15 (20 ng/ml), in the presence of anti-CD2/CD335 activator beads. **(G)** Quantification as in **(E)**, ΔgMFI values are CXCR4 stained cells minus isotype control. An unpaired t-test was applied, error bars ± SEM. **(H)** Western blot assessing P-ERK1,2^T202/Y204^ phosphorylation of untreated and CXCL12-stimulated (25 ng/ml, 5 min) CAR, CAR-CXCR4 and CAR-CXCR4^R334X^ NK-92 cells. Numbers between the gel lines give the ratios between P-ERK 1,2 and total ERK1,2. **(I)** Transwell migration assay assessing migratory capacity of CAR (grey bars) and CAR-CXCR4 (pink bars) NK-92 cells exposed to 6.25 ng/mL or 25 ng/mL CXCL12 for 4 hrs. Relative control (rel. ctrl.) represents unstimulated cells. Statistics calculated by an unpaired t-test, error bars ± SEM. **(J)** Quantification of migrated CAR (grey bar), CAR-CXCR4 (pink bar) and CAR-CXCR4^R334X^ (green bar) pNK cells exposed to 25 ng/mL CXCL12. Threshold set arbitrarily at 1.5-fold. Statistics calculated by an unpaired t-test, error bars ± SEM. P values are given. NK cells were used at day 12–15 after start of the culture. Data points in E, G, I, J represent independent biological replicates.

To assess if ectopically expressed CXCR4 coordinates chemotaxis of BCMA CAR-expressing NK cells towards CXCL12, we developed a BCMA CAR retroviral vector (CAR) (36) equipped with either a wildtype (WT), CXCR4 (CAR-CXCR4) or mutant CXCR4 (CAR-CXCR4^R334X^) chemokine receptor sequence (**Figure 1A**). The CXCR4^R334X^ sequence is a C-terminally-truncated, gain-of-function mutant with increased signal transduction, reduced desensitization, and impaired receptor internalization (37–41). Retroviral transduction of NK-92 cells with either the CAR, CAR-CXCR4 or CAR-CXCR4^R334X^ construct induced comparable levels of BCMA CAR expression (**Figures 1B, C**). Substantially higher levels of CXCR4 were detected on NK-92 cells transduced with the bicistronic CAR-CXCR4 or CAR-CXCR4^R334X^ constructs as compared to CAR NK-92 cells (**Figure 1D**). Furthermore, CXCR4 expression was greater on CAR-CXCR4^R334X^ NK-92 cells than CAR-CXCR4 NK-92 cells (**Figures 1D, E**).

Next, we generated CAR and CAR-CXCR4 primary NK cells (pNK; CD3^-^ CD56^+^) from peripheral blood (**Figure 1F**). These cells were transduced and expanded in the presence of IL-2 and IL-15 using (**Supplementary Figure S1C**) anti-CD2/CD355 MACS activator beads. On Day 9 (2-days post-transduction), gating on the CD56^+^CD16^high^ population, we observed substantially higher levels of CXCR4 expression on CAR-CXCR4 and CAR-CXCR4 R334X transduced pNK cells (**Figure 1F**). CXCR4 expression also increased modestly in CAR pNK cells, indicating endogenous CXCR4 upregulation was not induced by viral infection (12, 23, 41). Co-modified CAR-CXCR4 pNK cells expressed CXCR4 at two- to threefold higher levels (gMFI) when compared to pNK cells transduced with the conventional CAR (**Figures 1F, G**).

We functionally characterized the activity of ectopically-expressed CXCR4 by quantitating phosphorylation of downstream signaling modules in response to a 5 min CXCL12 exposure. CXCL12 addition triggered phosphorylation of p44/42 MAPK (ERK1,2) (**Figure 1H**; **Supplementary Figure S1D**) in CAR-CXCR4 and CXCR4^R334X^ NK-92 cells to a larger extent than in CAR NK-92 cells. We further confirmed AKT phosphorylation in CAR-CXCR4 NK-92 cells (**Supplementary Figures S1E, F**) after 5 min CXCL12 co-incubations (42).

CAR-CXCR4 co-expression led to fourfold improved chemotaxis towards CXCL12 (25 ng/ml) compared to NK-92 cells expressing the conventional CAR (**Figure 1I**). Along these lines, both CAR-CXCR4 and CAR-CXCR4^R334X^ pNK cells featured a higher migratory ability towards CXCL12 than CAR pNK cells (**Figure 1J**). For context, serum concentrations in MM patients are reportedly in the range of 453 ± 124 pg/mL (43). Despite a modest upregulation of endogenous CXCR4 in pNK cells (**Figure 1F**), they retained an inefficient ability to migrate towards CXCL12 at the concentrations indicated. Collectively, incorporation of either a WT or mutant CXCR4 sequence into a BCMA-CAR-encoding retroviral vector leads to the expression of a fully-functioning protein capable of instigating enhanced migration towards CXCL12 *in vitro*.

### Enforced CXCR4 expression enhances the cytolytic efficacy of CAR-NK cells *in vitro*

**Figure 2:**
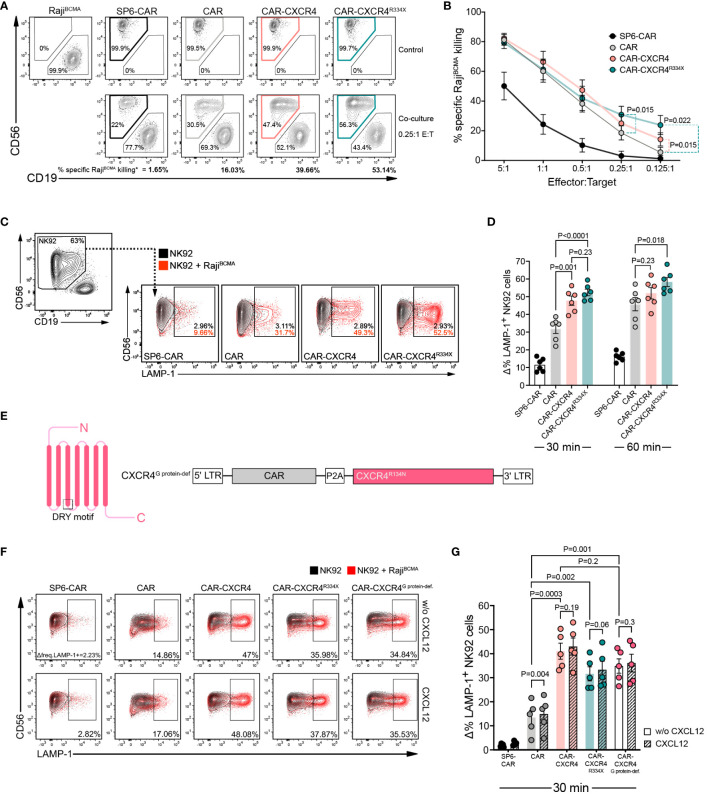
CXCR4 co-expression enhances cytolytic activity of BCMA-CAR NK cells in the absence of CXCL12 stimulation. **(A)** A flow cytometry-based cytotoxicity assay was performed by co-culturing CAR-transduced NK-92 cells and Raji^BCMA^ target cells. Representative FACS plot depicting a 17-hour co-culture between Raji^BCMA^ cells with either SP6-CAR as negative control, CAR, CAR-CXCR4 or CAR-CXCR4^R334X^ NK-92 cells at a 0.25:1 E:T ratio. Top panel displays Raji^BCMA^- or transduced NK-92-only controls. Bottom panel shows the co-cultures between Raji^BCMA^ cells (CD19^+^) and transduced-NK-92 cells (CD56^+^). The percentage of specific-Raji^BCMA^ cell killing is calculated using the formula: Killing rate %= (1- (tumor cells in coculture/tumor cells only)) x 100. (dependent upon E:T ratio). Numbers in the gates are the percentages of effector (CD56^+^) and target cells (CD19^+^), respectively. **(B)** Quantification of specific-Raji^BCMA^ cell killing (%) for the aforementioned co-cultures at the indicated E:T ratios (n=13–18 independent biological replicates). Statistics calculated by a paired t-test. **(C)** Representative FACS plot of a degranulation assay detecting LAMP-1 expression on CD56^+^ SP6-CAR as negative control, CAR, CAR-CXCR4 and CAR-CXCR4^R334X^ NK-92 cells following co-culture with Raji^BCMA^ cells for 30-min. Frequencies of LAMP-1^+^ NK-92 cells were calculated by subtracting percentage LAMP-1^+^ expression on transduced-NK-92 cells alone (grey plot) by percentage LAMP-1^+^ expression on NK-92 cells co-cultured with Raji^BCMA^ cells (red plot). Numbers within gates are the frequencies in percent of LAMP-1^+^ cells. **(D)** Quantification bar plots of **(C)** after 30- and 60-min stimulation. Statistics calculated by an unpaired t-test. **(E)** Schematic of G protein-deficient CXCR4^R134N^ mutant and retroviral construct. The position of the conserved DRY motif is indicated. **(F)** Representative FACS plot of a degranulation assay following a 30-minute co-culture between transduced NK-92 cells (including CAR-CXCR4^R134N^ mutant) and Raji^BCMA^ cells in the presence or absence of 25 ng/ml CXCL12. Numbers below gates are the frequencies in percent of LAMP-1^+^ cells upon stimulation. **(G)** Quantification of aforementioned degranulation assays (n=5) with (CXCL12; hatched bars) or without (w/o CXCL12; solid bars) CXCL12. Statistics calculated using an unpaired t-test comparing different constructs and a paired t-test assessing each construct with or without CXCL12. Error bars ± SEM. P values are given; data points in **(D)**, **(G)** represent independent biological replicates.

Previous reports in T cells have shown that recruitment of chemokine receptors within the IS enhances TCR-induced signaling and IS longevity (33, 35). Canonically, these co-stimulatory effects have been attributed to a chemokine stimulus acting on the cognate chemokine receptor. However, the conditions for non-conventional CAR-dependent signaling processes in NK cells are unknown. Hence, we first investigated whether enforced CXCR4 expression boosts the antigen-dependent cytolytic efficacy of BCMA CAR-NK cells. CAR, CAR-CXCR4, CAR-CXCR4^R334X^ and SP6-control (irrelevant CAR binder) NK-92 cells were co-cultured with an engineered BCMA-expressing Raji target cell line (Raji^BCMA^) (**Supplementary Figure S2**). The proportion of viable Raji^BCMA^ cells remaining in culture after 17 hours was quantified by flow cytometry and showed that CAR-CXCR4 and CAR-CXCR4^R334X^ NK-92 cells possessed a 15–25% higher antigen-dependent killing capacity compared to CAR NK-92 cells at lower effector-to-target ratios (E:T: 0.125:1) (**Figures 2A, B**). To exclude effector cell type-specific peculiarities, we conducted analogous experiments with the NK cell line YTS (44) (**Supplementary Figure S3A**). Indeed, CXCR4-equipped CAR YTS cells exhibited a higher killing capacity against Raji^BCMA^ cells compared to conventional CAR YTS cells (**Supplementary Figures S3B, C**).

We next performed a CD107a (LAMP-1) secretory lysosome degranulation assay to scrutinize whether CXCR4 overexpression endows BCMA CAR-NK-92 cells with enhanced antigen-dependent cytolytic efficacy (45, 46). Following 30 min co-cultures with Raji^BCMA^ cells, we detected substantially higher levels (approximately 20%) of LAMP-1 expression on the surfaces of CAR-CXCR4 and CAR-CXCR4^R334X^ NK-92 cells (**Figures 2C, D**). This difference was diminished after 60 min, indicating that CXCR4 supports a rapid mobilization of lytic granules following CAR activation (**Figure 2D**).

Whereas previous reports have postulated a synergistic, mutually-reciprocal state of TCR- and chemokine-induced receptor activation (47, 48), others have alluded to a state whereby CXCL12-induced signals do not override those emanating from TCR activation (49, 50). Consistent with the latter, we found that CXCL12 supplementation did not enhance antigen-dependent degranulation among CAR-CXCR4 or CAR-CXCR4^R334X^ NK-92 cells (**Figures 2E–G**). This was, in part, because CAR NK cells lacking CXCR4 overexpression exhibited similar low-level gain-of-function (**Figures 2F, G**). We also generated a CXCR4 mutant with a defect in G protein coupling (R134N) in the conserved DRY motif (CAR-CXCR4^G protein-def.^) (**Figures 2E–G**). This mutation does not affect downstream PI3K-activation, tyrosine phosphorylation of focal adhesion proteins and chemotaxis (51). When linked to the BCMA CAR in our bicistronic constructs, we confirmed that CAR-CXCR4, CAR-CXCR4^R334X^ and CAR-CXCR4^G protein-def.^ NK-92 cells maintained a superior antigen-dependent degranulation capacity (**Figures 2F, G**). Our observations imply a hierarchy in which the enhanced killing capacity originates from CAR-driven activation of CXCR4 signaling nodes.

### CXCR4-equipped CAR NK-92 cells are efficacious *in vitro* against MM cell lines and primary patient-derived MM cells

**Figure 3:**
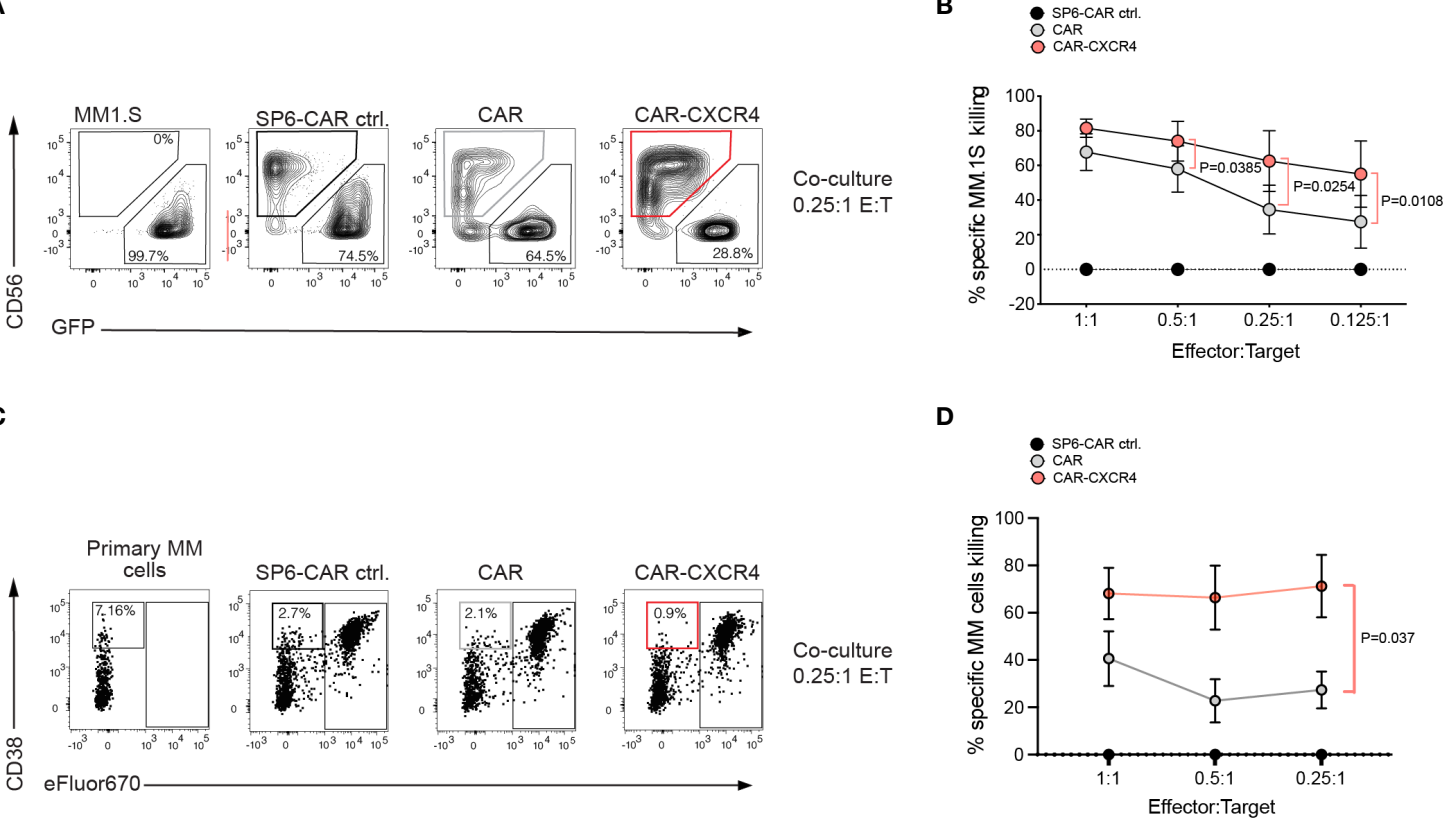
Multiple Myeloma cells with endogenous BCMA expression are more susceptible to BCMA CAR NK-92 cells equipped with the CXCR4 chemokine receptor. **(A)** A flow cytometry-based cytotoxicity assay was performed by co-culturing CAR-transduced NK-92 cells and MM.1S-luc.GFP target cells. Representative FACS plot depicting a 6-hour co-culture between MM.1S-luc.GFP cells with either SP6-CAR as negative control, CAR, or CAR-CXCR4 NK-92 cells at a 0.25:1 E:T ratio. Effector NK-92 cells were detected by anti-CD56 staining, and target cells were GFP labeled for discrimination. Numbers on the bottom gates are percentages of viable tumor target cells after co-culture. **(B)** The percentage of specific-MM.1S-luc.GFP cell killing is calculated as in **Figure 2A**). (dependent upon E:T ratio). Values displayed are mean ± SEM (n=4 independent experiments). Statistics calculated by a paired t-test. **(C)** A representative FACS plot of co-cultures between primary MM specimen and NK-92 effector cells. Effector cells were labeled with eFluor670^+^ for better distinction from MM target cells, defined by CD38^+high^ eFluor670^-^. Because of the low frequency of primary MM target cells in the specimen, killing rates were calculated relative to the negative control, SP6 CAR. Statistics calculated by a paired t-test; n=4 independent donor samples tested. **(D)** Formula to quantify the killing rate is Killing rate %= (1- (tumor cells in coculture with test CAR/tumor cells in coculture with SP6 CAR control)) x100.

To rule out an artifact of enhanced BCMA expression in the transfected Raji^BCMA^ cell line, we compared the antitumor cytolytic activity of CAR and CAR-CXCR4 NK-92 in co-cultures with the MM cell line MM.1S and with four patient-derived primary MM specimen. In agreement with the improved cytolytic activity against Raji^BCMA^ cells, CXR4 co-expression endowed CAR NK-92 cells with an enhanced cytolytic capacity against both cell types exhibiting endogenous BCMA expression (**Figures 3A–D**). CAR-CXCR4 NK-92 cells gained a 25% higher antigen-dependent killing capacity for MM.1S cells compared to CAR NK-92 cells, visible at lower effector-to-target ratios (E:T: 0.125:1) (**Figure 3B**). The FACS-based killing assay was corroborated by a degranulation assay, using the MM.1S cell line as a stimulus (**Supplementary Figures S4A, B**). Similar to the MM.1S cell line, CXCR4 co-expression endowed CAR NK-92 cells with much stronger cytolytic capacity against primary MM cells (**Supplementary Figure S4C**) at low effector to target ratios (E:T: 0.25:1), which even exceeded the gain-of-function compared to Raji^BCMA^ and MM.1S cells (**Figures 3C, D**). Notably, the frequencies of MM cells within patient-derived primary bone marrow aspirates are much lower than homogenous target cell populations as for Raji^BCMA^ and MM.1S cell lines.

To exclude a CAR-independent response of chemokine receptor overexpression, we also generated NK-92 cells expressing CXCR4 only. We studied the innate killing capacity of CXCR4 NK-92 cells in a co-culture with MM.1S cells. Isolated CXCR4 overexpression was not sufficient to endow NK-92 cells with enhanced degranulation capacity. Their innate reactivity towards MM.1S tumor cells was similar to NK-92 cells only (mean LAMP-1 upregulation: 2.3%; CXCR4 NK-92: mean 3.4%), and substantially weaker than CAR NK-92 cells. This result indicated that the gain of cytotoxic capacity in NK-92 cells depends on a co-stimulatory function of the chemokine receptor in the process of BCMA CAR activation (**Supplementary Figures S4D–F**).

### CAR-CXCR4 co-expression prolongs CAR surface expression and augments trogocytosis

**Figure 4:**
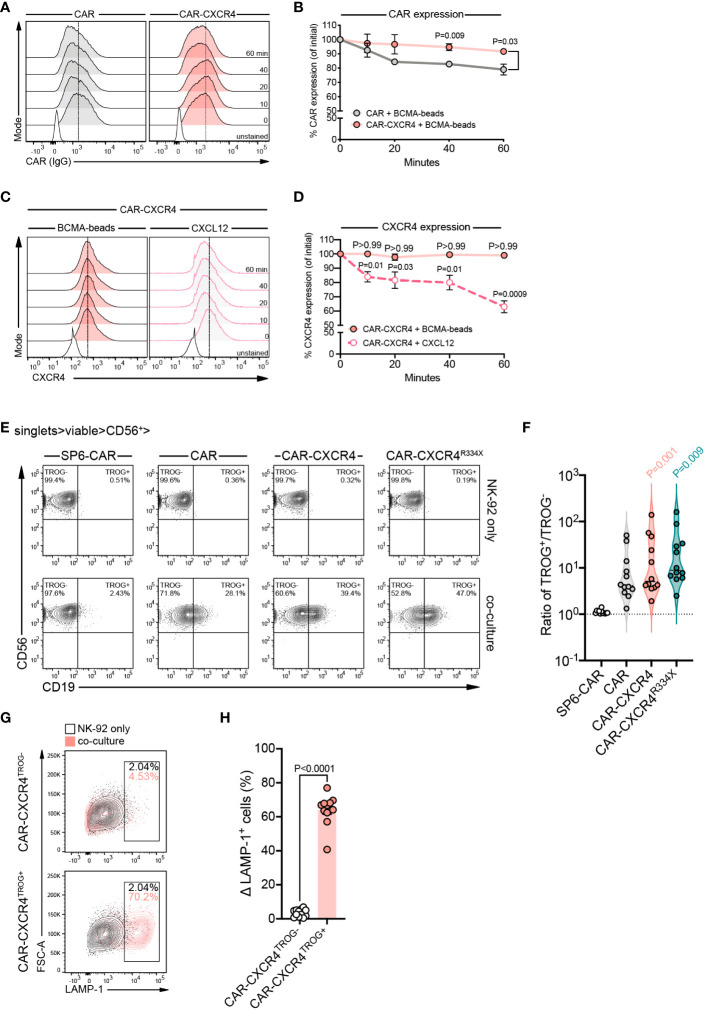
CXCR4 overexpression prolongs BCMA-CAR surface expression and is associated with enhanced levels of CAR-mediated trogocytosis. **(A)** Representative FACS histograms showing the kinetics of BCMA-CAR surface expression (as detected by IgG staining) on CAR (grey histograms) and CAR-CXCR4 (pink histograms) NK-92 cells following exposure to BCMA-beads for the indicated timepoints. **(B)** Quantification of BCMA-CAR surface expression. gMFI values were set arbitrarily at 100% at 0 min. The change in gMFI over time was quantified as a percentage of initial surface expression (n=3 independent biological replicates). Statistics calculated using an unpaired t-test (CAR versus CAR-CXCR4). **(C)** Representative FACS histograms of CXCR4 expression on CAR-CXCR4 NK-92 cells after exposure to BCMA-beads (pink histograms) or CXCL12 (grey histograms; pick-dotted line) for the indicated timepoints. **(D)** Quantification of CXCR4 surface expression, calculated as for **(B)** (BCMA-beads n=4; CXCL12 n=5 independent biological experiments). Statistics calculated using a paired t-test (BCMA-beads; timepoint versus ‘0 min’) or Wilcoxon test (CXCL12; timepoint versus ‘0 min’), where appropriate according to sample size and distribution. **(E)** Representative FACS plots depicting the proportion (%) of CD56^+^ CD19^+^ SP6-CAR, CAR, CAR-CXCR4 and CAR-CXCR4^R334X^ NK-92 cells (ordinarily CD19^-^) following 30 min co-culture with CD19^+^ Raji^BCMA^ target cells, indicative of CAR activation-induced trogocytosis. Cell gating was on single cells (FSC-W/FSC-H). Top panel shows ‘NK-92 only’ control cells, while the bottom panel represents the co-culture. **(F)** Violin plots quantifying the ratio of SP6-CAR (white plot), CAR (grey), CAR-CXCR4 (pink) and CAR-CXCR4^R334X^ (green) NK-92 cells that had undergone (TROG+) or had not yet experienced (TROG-) trogocytosis of Raji^BCMA^ target cells (TROG+/TROG-). Individual datapoints for independent biological replicates are shown. Statistics calculated using a paired t-test. **(G)** Representative FACS plots detecting LAMP-1 expression on the aforementioned CAR-CXCR4^TROG-^ (CD56^+^ CD19^-^; top panel) and CAR-CXCR4^TROG+^ (CD56^+^ CD19^+^; bottom panel) NK-92 cells following a 30 min co-culture with Raji^BCMA^ cells. Frequencies of LAMP-1^+^ CAR-CXCR4^TROG-^ or CAR-CXCR4^TROG+^ cells were calculated by subtracting percentage LAMP-1^+^ expression on CAR-CXCR4 NK-92 cells alone (black contour plot) by percentage LAMP-1^+^ expression on either CAR-CXCR4^TROG-^ or CAR-CXCR4^TROG+^ NK-92 cells (pink contour plot). Numbers within gates are the frequencies in percent of LAMP-1^+^ cells. **(H)** Quantification bar plots of **(G)**. Data points represent independent biological replicates. Statistics calculated by a paired t-test. P values are given; statistical significance P<0.05. Error bars ± SEM.

To dissect the mechanisms underlying the increased cytolytic activity of CAR-CXCR4 NK-92 cells, we focused on CAR stabilization and the initiation of proximal or distal signaling. Because reorganization and accumulation of chemokine receptors within the IS of conventional T cells with antigen-presenting cells (APC) has been shown to stabilize the IS (33, 35), we explored whether ectopic CXCR4 overexpression affects BCMA-CAR internalization during antigen-engagement. CAR and CAR-CXCR4 NK-92 cells were exposed to cognate BCMA peptide-coated magnetic beads (BCMA-beads) and examined for the surface expression of the BCMA-CAR over-time. BCMA-beads offer the operational advantage of selectively binding to the BCMA-CAR cognate receptor. Although both CAR and CAR-CXCR4 NK-92 cells progressively lost BCMA-CAR surface expression after BCMA-bead exposure, CAR-CXCR4 NK-92 cells prolonged BCMA-CAR surface expression to a greater extent than CAR NK-92 cells (**Figures 4A, B**). BCMA-bead stimulation did not affect CXCR4 surface expression, but on the contrary, CXCL12 induced internalization of its cognate receptor (**Figures 4C, D**). These observations suggest that the integrity of the BCMA CAR-driven IS is maintained in the presence of abundant CXCR4. Exposing CAR- and CAR-CXCR4 NK-92 cells to BCMA-beads produced no striking difference in IFN-γ secretion (**Supplementary Figure S5**).

Recently, trogocytosis induced by CAR-activation on NK cells was associated with higher levels of degranulation towards target cells (52). To assess the degree of trogocytosis induced by CAR engagement, we quantified the ratio between CD56^+^/CD19^+^ double-positive (TROG^+^) and CD56^+^/CD19^-^ (TROG^-^) BCMA CAR-expressing NK-92 cells in response to CD19^+^ Raji^BCMA^ target cell exposure. CAR-CXCR4 (mean TROG^+^/TROG^-^=1.04 ± 0.59) and CAR-CXCR4^R334X^ (1.17 ± 0.53) NK-92 cells triggered trogocytosis to a substantially higher degree than CAR NK92 cells (0.79 ± 0.47) (**Figures 4E, F**). We then asked whether trogocytosis modulated effector function of CAR-CXCR4 NK-92 cells by assessing the level of degranulation. Whereas minimal levels of LAMP-1 expression were detected on CAR-CXCR4^TROG-^ NK92 cells (mean % ΔLAMP-1+ cells= 3.39 ± 2.14), a larger proportion of CAR-CXCR4^TROG+^ NK92 cells had undergone degranulation (64.02 ± 8.74) (**Figures 4G, H**). Prolonged expression of the BCMA CAR at the cell surface and higher levels of trogocytosis following target cell challenge suggest that CAR-CXCR4 co-modification equips NK92 cells with a substantially enhanced effector function.

### Engineered CXCR4 expression boosts BCMA-induced proximal signaling by enhanced ZAP-70 activation

**Figure 5:**
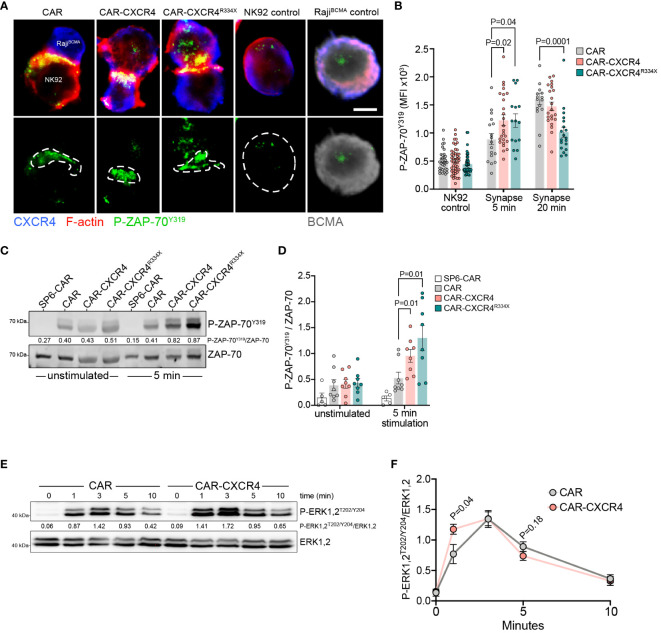
Enforced CXCR4 expression is associated with increased P-ZAP-70^Y319^ recruitment into the IS and boosted BCMA-induced proximal signaling. **(A)** Exemplary single plane micrographs depicting IS formation between Raji^BCMA^ cells and CAR, CXCR-CXCR4 or CAR-CXCR4^R334X^ NK-92 cells during 5 min co-incubations. The immunological synapse was identified by accumulation of actin cytoskeleton at the interface of NK-92 cells and Raji^BCMA^ target cells. Cells were probed for CXCR4 (blue), P-ZAP-70^Y319^ (green), F-actin (red; representative of IS interface) and BCMA (grey; target cell marker). The region of interest (ROI) represents the IS, where P-ZAP-70^Y319^ localizes to. Scale bar, 5 µm. Representative image, acquired on an LSM-980 airyscan microscope, equipped with a 63x Plan Apochromat NA 1.40 oil objective. Digital images were obtained with ZEN software (Zeiss), and further processed with Fiji. **(B)** Quantification of phosphorylated P-ZAP-70^Y319^ MFI within the ROI following 5- or 20-minute co-incubations. CAR=grey bar, CAR-CXCR4=pink bar and CAR-CXCR4^R334X^=green bar. Statistics calculated using an unpaired t-test. Each data point represents one effector-target NK-92-Raji^BCMA^ cell conjugate. At least n=2 independent experiments were performed. **(C)** Representative immunoblot assessing P-ZAP-70^Y319^ phosphorylation of unstimulated and BCMA-bead-stimulated (5-minute) SP6-CAR control, CAR, CXCR-CXCR4 and CAR-CXCR4^R334X^ NK-92 cells. **(D)** Quantification (densitometry) of P-ZAP-70^Y319^ phosphorylation for the aforementioned Western blots (n=8 independent biological replicates), calculated by dividing P-ZAP-70^Y319^ signal intensity by total ZAP-70 signal intensity. SP6-CAR control=white bars, CAR=grey bars, CAR-CXCR4=pink bars, and CAR-CXCR4^R334X^=green bars. Individual datapoints shown. Statistics calculated using a Mann-Whitney test. **(E)** Kinetics of ERK1,2 phosphorylation assessed by immunoblotting of BCMA-bead stimulated CAR-transduced NK-92 cells. In C) and E), numbers between the gel lines give the ratio between phosphorylated ZAP-70 and phosphorylated ERK 1,2 and their total protein forms ERK1,2 or AKT, respectively. **(F)** Quantification of P-ERK1,2^T202/Y204^ occurrence. The ratio of total ERK1,2 and phosphorylated P-ERK1,2 is given (n=6 independent biological replicates). Statistics calculated using an unpaired t-test. Error bars ± SEM.

CAR engagement results in the formation of a nonclassical, disorganized IS characterized by the rapid initiation of proximal signaling, and quicker recruitment of cytotoxic granules (53–55). It was recently determined that the sensitivity of a CAR-T cell towards antigen is approximately 1000-times reduced compared to that of conventional antiviral T cells, at least in part due to inefficient recruitment of ZAP-70 to the engaged CAR (56). Considering that chemokine receptor signal transduction relies on ZAP-70 (48, 57) recruitment and furthermore, promotes chemokine-enrichment in T cell synapses, we asked whether enforced CXCR4 during CAR engagement would augment ZAP-70 recruitment. We visualized the presence of phosphorylated ZAP-70 (P-ZAP-70) within the synapse of engaged CAR, CAR-CXCR4 and CAR-CXCR4^R334X^ NK-92 cells following a short co-culture with Raji^BCMA^ cells (**Figures 5A, B**). Thereafter, we quantified the expression (MFI) of P-ZAP-70 within IS. These data showed that P-ZAP-70 expression was significantly higher within the synapse of CAR-CXCR4 and CAR-CXCR4^R334X^ NK-92 cells compared to CAR-NK-92 cells, which was most pronounced after 5 min (**Figure 5B**). After 20 min, synaptic P-ZAP-70 MFI values in CAR NK-92 cells were comparable to those obtained in CAR-CXCR4 NK-92 cells, further suggesting that CXCR4 supports the initial events triggered by CAR engagement. Of note, CXCR4 expression in Raji^BCMA^ target cells interfered with the quantitation of synaptic CXCR4 recruitment in APC-conjugated CAR NK cells.

Immunoblot analysis of protein lysates derived from short-term (5 min) BCMA-bead stimulated NK-92 cells revealed a significant enhancement of pZAP-70 expression in CAR-CXCR4 and CAR-CXCR4^R334X^ modified NK-92 cells (**Figures 5C, D**). We further inspected the signaling kinetics of antigen-engagement over-time by comparing induction of ERK1,2, which occurs downstream of ZAP-70 activation. After only one min exposure to BCMA-beads, ERK1,2 phosphorylation was significantly increased in CAR-CXCR4 NK-92 cells compared to CAR NK-92 cells. We conclude that BCMA-CAR activation is more potent when NK-92 cells are equipped with CXCR4 (**Figures 5E, F**).

### Enforced CXCR4 expression lowers the antigen amount necessary for BCMA-CAR activation

**Figure 6:**
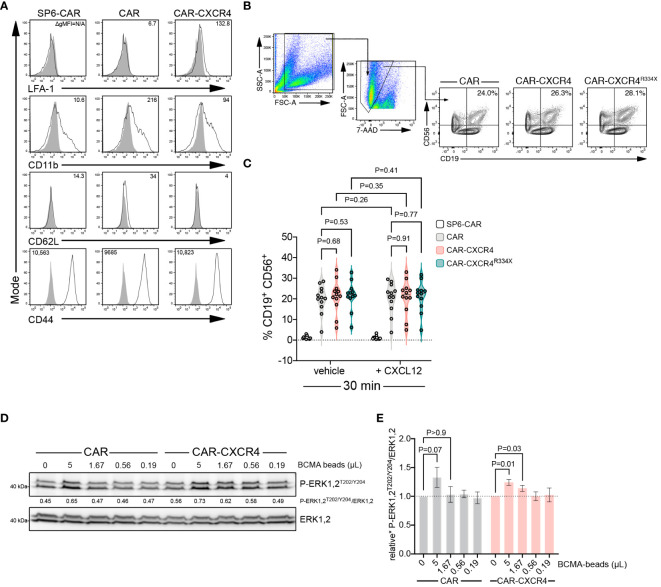
Enforced CXCR4 expression lowers the antigen threshold for BCMA-CAR activation and heightens BCMA-CAR sensitivity. **(A)** Flow cytometry analysis of adhesion molecule expression in BCMA-CAR and CAR-CXCR4 enhanced NK-92 cells. Numbers in the histograms indicate gMFI values, grey solid graphs depict isotype controls. **(B)** CAR-transduced NK-92 cells and Raji^BCMA^ target cells were co-cultured as in **Figures 2C, F**. Conjugate formation was performed either with or without CXCL12 supplementation (25 ng/ml). After 30 min, cells were stained for CD56 and CD19. Numbers in the gates indicate the percentage of cells forming conjugates. **(C)** Quantification of conjugate formation, as determined by cell doublets (FSC-H/FSC-W) and CD56^+^/CD19^+^ double positive cells. SP6-CAR ctrl=white plot, CAR=grey plot, CAR-CXCR4=pink plot, and CAR-CXCR4^R334X^=green plot. Individual datapoints for biological replicates are shown. An unpaired and paired t-test was applied, where appropriate. **(D)** Representative immunoblot examining rapid P-ERK1,2^T202/Y204^ phosphorylation of CAR and CXCR-CXCR4 NK-92 cells exposed to decreasing amounts of cognate BCMA antigen (BCMA-beads; 5, 1.67, 0.56, 0.19 and 0 µL) for 5 minutes. **(E)** Quantification (densitometry) of P-ERK1,2^T202/Y204^ phosphorylation for the abovementioned western blots (n=7 independent biological replicates), calculated by P-ERK1,2^T202/Y204^ signal intensity divided by ERK1,2 signal intensity. CAR=grey bars and CAR-CXCR4=pink bars. Statistics calculated using a Wilcoxon test.

CXCR4 expression could influence the formation of mature IS by enabling increased integrin inside-out (33) signaling. Using a conjugate formation assay we quantitated stable IS formation between CAR NK effector cells and Raji^BCMA^ target cells. All NK-92 CAR constructs were devoid of integrin LFA-1 (open conformation) and adhesion molecule CD62L, but ubiquitously expressed CD11b and CD44 (**Figure 6A**). Conjugate formation after 30 min was indistinguishable between those cell types, addition of CXCL12 had no stimulatory impact in this process (**Figures 6B, C**). Potentially, conjugate formation could occur earlier than 30 min, which would be consistent with an earlier activation of the signaling cascade when CXCR4 is overexpressed. This was not further investigated herein.

Inadequate CAR reactivity towards low-density cognate antigen is a major source of treatment failure (58). Insufficient recruitment of ZAP-70 to the engaged CAR has emerged as an important cause of reduced CAR sensitivity (56). Because enforced CXCR4 expression enhances the presence of activated ZAP-70 within the IS (**Figures 5A–D**), we examined whether the presence of CXCR4 equips BCMA-CAR NK cells with an improved ability to recognize target cells with lower antigen amounts. We exposed CAR and CAR-CXCR4 NK-92 cells to lowering amounts of BCMA-beads and assessed the induction of downstream ERK1,2 signaling. CAR-CXCR4 NK-92 cells recognized and transmitted signals when antigen availability was lower compared to its conventional CAR counterpart (**Figures 6D, E**). More specifically, at a BCMA-bead concentration of 5 and 1.67 µL, CAR-CXCR4 NK-92 cells were still able to trigger ERK1,2 phosphorylation (**Figure 6D**). These data suggest that enhanced ZAP-70 recruitment at the IS, afforded by the presence of synthetic CXCR4, would enable CAR-CXCR4 NK-92 cells to eliminate lower-antigen-expressing tumor cells.

## Discussion

The findings reported herein demonstrate that engineered overexpression of CXCR4 bestows BCMA CAR-NK-92 cells with an increased ability to eliminate BCMA-expressing tumor cells *in vitro*. We show that CXCR4 possesses additional functions beyond its classical role of coordinating cell migration towards CXCL12 gradients. In fact, linked to the redistribution of CAR-T and CAR-NK cells into the tumor niche, reports have demonstrated that gains in anti-tumor efficacy are merely due to locally augmented effector cell populations. Indeed, enhancing homing capacity has been shown to enrich their accumulation within malignant tissues and, consequently, elicit tumor suppression of solid cancers (18–20), and hematological malignancies (16, 23, 32). However, pertaining to the co-stimulatory effects of chemokine receptors in TCR-mediated T cell activation (35, 47, 59), we also argue that outfitting BCMA CAR-expressing NK-92 cells with CXCR4 endows these cells with an enhanced ability to kill BCMA antigen-expressing tumor cells on a cellular level.

The degree to which chemokine receptors play a co-stimulatory role during T cell activation without their chemokine ligand (59) has garnered some debate. On the one hand, studies have shown that recruitment of CXCR4, CCR5 (35) and CCR7 (33) into the IS after TCR-engagement relies on chemokine ligand binding, as chemokine receptor enrichment within the T cell-APC contact site was reduced in the absence of chemokine ligands. CXCL12 has been suggested to prompt the physical association of CXCR4 with the TCR. The resultant recruitment of ZAP-70 *via* SH2-domain binding to TCR-associated phospho-ITAMs prolonged ERK kinase activity, intracellular Ca^2+^ signaling and cytokine secretion (60). Thus, it would appear that chemokine receptor triggering on T cells is a precondition for chemokine receptor-mediated co-stimulation. On the other hand, Felce et al. demonstrated that CXCR4 organization within the IS was not influenced by CXCL12 supplementation, nor did CXCR4 form complexes with the TCR (49). Kremer et al. and Dinkel et al. collectively showed that TCR crosslinking transactivated CXCR4 in the absence of CXCL12 *via* GRK2-induced phosphorylation of the cytoplasmic CXCR4^S339^ residue. The latter was in turn shown to highjack PREX1-Rac1 signaling to augment cytokine secretion (20, 61). Hence accumulative data suggest that CXCR4 activation and transactivation can occur irrespective of CXCL12 binding and downstream of TCR ligation.

Here, we provide multiple lines of evidence that demonstrate the enhanced anti-myeloma effects of CXCR4 co-modified BCMA CAR-NK cells in a chemokine-independent manner. Firstly, we co-cultured BCMA CAR-transduced NK-92 or YTS effector cells with target cells in the absence of exogenous chemokine, revealing that CXCR4-expressing CAR-NK cells feature elevated cytotoxic activity. Secondly, degranulation assays further supported the notion that CXCR4 acts as a co-receptor during CAR-engagement by triggering rapid activation and cytolytic efficacy in NK-92 cells, independent of CXCL12. Thus, CXCL12 does not override TCR-induced (49, 50), and, as evidenced in this study, CAR-induced signaling. To rule out the possibility that EBV-transformed Raji^BCMA^ target cells provoke CXCL12 secretion (62) or contribute towards other NK stimulatory signals, we used BCMA-peptide-decorated microbeads. Antigen-independent stimuli involve integrin conjugation or Fc-receptor signaling that can confound BCMA CAR-dependent signaling. BCMA-beads stimulated CAR activation in a strictly antigen-dependent and chemokine-independent manner. Our data demonstrating the accelerated kinetics of ERK1,2 and ZAP-70 phosphorylation in CXCR4-expressing NK-92 cells are in line with enhanced BCMA CAR activation, which results in increased cytotoxic capacity *in vitro*.

How can we reconcile a CXCR4-dependent, but CXCL12-independent, co-stimulatory role of this receptor with previous reports on ligand-dependent CXCR4, CCR5 or CCR7 functionality in TCR-expressing T cells? To our knowledge, the mechanistic interplay between CARs and cooperating chemokine receptors have not been addressed in NK cells. Furthermore, fundamental differences exist between the formation of TCR- and CAR-induced synapses. Reports have demonstrated that CAR-antigen complexes form disorganized synapses in which the architecture deviates from a TCR-pMHC synapse by lack of defined central, peripheral and distal supramolecular activation complexes. Indeed, organized TCR-antigen synapses allow for a lower threshold for antigen recognition (53, 63). In fact, although CARs outperform TCRs with regard to antigen binding, proximal signaling in the CAR synapse is strongly attenuated, which results in reduced responsiveness to low antigen levels.

This effect appears to result from insufficient ZAP-70 recruitment to ligated CARs, followed by decreased signal activation. Importantly, low responsiveness facilitates immune escape, which has been reported for CD19 and CD22 CAR-T cell therapies (64–66). In our report, we revealed an enhanced accumulation of P-ZAP-70 within the synapse of CXCR4-expressing BCMA CAR-NK-92 cells, indicating that activation of ZAP-70 at the synapse is accelerated. Cycling and activation of ZAP-70 is integral to signal amplification and Ca^2+^ fluxing, resulting in the induction of downstream signaling cascades (67, 68). Together, CXCR4 can partially compensate for the drawbacks associated with CAR-induced synapses to foster detection of antigen at lower levels given earlier and more profound ZAP-70 activation.

CXCR4-equipped NK cells were able to activate the ERK1,2 signaling pathway at significantly lower microbead-bound antigen amounts than BCMA CAR NK cells. It seems plausible that BCMA-CAR sensitivity towards antigen can be fine-tuned by CXCR4 overexpression. Moreover, CAR-triggered signaling is more rapid, and consequently, cytotoxic granule release at the synapse followed by target cell killing is faster (53). Although the mechanistic link between the initial signal (the CAR-antigen encounter) and chemokine-independent CXCR4 co-stimulation remains to be further explored, it is conceivable that crosstalk induced by activated ZAP-70 may affect signal strength and onset.

TCR deubiquitination impairs receptor downregulation, which consequently promotes enhanced T cell responses (69, 70). As CARs emulate much of TCR-proximal signaling, it has been suggested that CAR downmodulation might attenuate tumor cell killing capacity (71, 72). Our results support the notion that counteracting CAR downmodulation via CXCR4-co-expression may improve overall CAR-T effector functions, a matter which has to our knowledge not been addressed in NK cells. We consider it likely that extended surface residency delays endocytosis and lysosomal degradation (73).

Collectively, we suggest that the mechanisms underlying this observation involves (i) CXCR4-mediated P-ZAP-70 recruitment into the synapse, (ii) the stabilization of the synapse and CAR surface deposition, and (iii) intensified antigen-dependent CAR activation by hijacked or colluding signaling nodes. We propose that the homeostatic chemokine receptor CXCR4 acts as a co-receptor during the formation of a CAR-mediated IS, which gains relevance in NK cells that have endogenously low CXCR4 expression.

## Data availability statement

The datasets presented in this article are not readily available because data generation only refers to *in vitro* data from common cell lines. For primary MM samples, patient data are anonymized and not visible or available to the researchers, no further storage of data derived thereof. Requests to access the datasets should be directed to arehm@mdc-berlin.de.

## Ethics statement

The studies involving humans were approved by Ethic Committee at Charite University Medicine Berlin, No. EA2/142/20; EA2/216/18. The studies were conducted in accordance with the local legislation and institutional requirements. The participants provided their written informed consent to participate in this study.

## Author contributions

MWM: Data curation, Formal analysis, Investigation, Methodology, Visualization, Writing – original draft. HE: Investigation, Methodology, Resources, Writing – review & editing. LM: Formal analysis, Investigation, Methodology, Visualization, Writing – review & editing. MM: Investigation, Methodology, Writing – review & editing. AF: Investigation, Methodology, Writing – review & editing. MB: Methodology, Writing – review & editing. MS: Investigation, Methodology, Writing – review & editing. KG: Investigation, Methodology, Writing – review & editing. VG: Investigation, Methodology, Writing – review & editing. JR: Resources, Supervision, Writing – review & editing. KM: Supervision, Writing – review & editing. JG: Supervision, Writing – review & editing. JH: Methodology, Resources, Writing – review & editing. JK: Resources, Writing – review & editing. BV: Resources, Supervision, Writing – review & editing. UH: Conceptualization, Formal analysis, Funding acquisition, Methodology, Project administration, Resources, Supervision, Writing – review & editing. AR: Conceptualization, Formal analysis, Funding acquisition, Investigation, Methodology, Project administration, Resources, Supervision, Validation, Writing – review & editing.

## References

[1] Mikkilineni L Kochenderfer JN. CAR T cell therapies for patients with multiple myeloma. Nat Rev Clin Oncol. (2021) 18:71–84. doi: 10.1038/s41571-020-0427-6 32978608

[2] van de Donk N Usmani SZ Yong K. CAR T-cell therapy for multiple myeloma: state of the art and prospects. Lancet Haematol. (2021) 8:e446–e61. doi: 10.1016/S2352-3026(21)00057-0 34048683

[3] Munshi NC Anderson LD Jr. Shah N Madduri D Berdeja J Lonial S. Idecabtagene vicleucel in relapsed and refractory multiple myeloma. N Engl J Med. (2021) 384:705–16. doi: 10.1056/NEJMoa2024850 33626253

[4] Martin T Usmani SZ Berdeja JG Agha M Cohen AD Hari P. Ciltacabtagene autoleucel, an anti-B-cell maturation antigen chimeric antigen receptor T-cell therapy, for relapsed/refractory multiple myeloma: CARTITUDE-1 2-year follow-up. J Clin Oncol. (2023) 41:1265–74. doi: 10.1200/JCO.22.00842 PMC9937098 35658469

[5] Berdeja JG Madduri D Usmani SZ Jakubowiak A Agha M Cohen AD. Ciltacabtagene autoleucel, a B-cell maturation antigen-directed chimeric antigen receptor T-cell therapy in patients with relapsed or refractory multiple myeloma (CARTITUDE-1): a phase 1b/2 open-label study. Lancet. (2021) 398:314–24. doi: 10.1016/S0140-6736(21)00933-8 34175021

[6] Zhang X Zhang H Lan H Wu J Xiao Y. CAR-T cell therapy in multiple myeloma: Current limitations and potential strategies. Front Immunol. (2023) 14:1101495. doi: 10.3389/fimmu.2023.1101495 36891310 PMC9986336

[7] Pont MJ Hill T Cole GO Abbott JJ Kelliher J Salter AI. gamma-Secretase inhibition increases efficacy of BCMA-specific chimeric antigen receptor T cells in multiple myeloma. Blood. (2019) 134:1585–97. doi: 10.1182/blood.2019000050 PMC6871311 31558469

[8] Fernandez de Larrea C Staehr M Lopez AV Ng KY Chen Y Godfrey WD. Defining an optimal dual-targeted CAR T-cell therapy approach simultaneously targeting BCMA and GPRC5D to prevent BCMA escape-driven relapse in multiple myeloma. Blood Cancer Discov. (2020) 1:146–54. doi: 10.1158/2643-3230.BCD-20-0020 PMC7575057 33089218

[9] Abecassis A Roders N Fayon M Choisy C Nelson E Harel S. CAR-T cells derived from multiple myeloma patients at diagnosis have improved cytotoxic functions compared to those produced at relapse or following daratumumab treatment. EJHaem. (2022) 3:970–4. doi: 10.1002/jha2.479 PMC9421998 36051036

[10] Themeli M Riviere I Sadelain M. New cell sources for T cell engineering and adoptive immunotherapy. Cell Stem Cell. (2015) 16:357–66. doi: 10.1016/j.stem.2015.03.011 PMC5611846 25842976

[11] Laskowski TJ Biederstadt A Rezvani K. Natural killer cells in antitumour adoptive cell immunotherapy. Nat Rev Cancer. (2022) 22:557–75. doi: 10.1038/s41568-022-00491-0 PMC9309992 35879429

[12] Liu E Tong Y Dotti G Shaim H Savoldo B Mukherjee M. Cord blood NK cells engineered to express IL-15 and a CD19-targeted CAR show long-term persistence and potent antitumor activity. Leukemia. (2018) 32:520–31. doi: 10.1038/leu.2017.226 PMC6063081 28725044

[13] Cichocki F Bjordahl R Goodridge JP Mahmood S Gaidarova S Abujarour R. Quadruple gene-engineered natural killer cells enable multi-antigen targeting for durable antitumor activity against multiple myeloma. Nat Commun. (2022) 13:7341. doi: 10.1038/s41467-022-35127-2 36446823 PMC9709157

[14] Tang X Yang L Li Z Nalin AP Dai H Xu T. First-in-man clinical trial of CAR NK-92 cells: safety test of CD33-CAR NK-92 cells in patients with relapsed and refractory acute myeloid leukemia. Am J Cancer Res. (2018) 8:1083–9. PMC6048396 30034945

[15] Grzywacz B Moench L McKenna D Jr. Tessier KM Bachanova V Cooley S. Natural killer cell homing and persistence in the bone marrow after adoptive immunotherapy correlates with better leukemia control. J Immunother. (2019) 42:65–72. doi: 10.1097/CJI.0000000000000250 30489431 PMC6365204

[16] Ng YY Du Z Zhang X Chng WJ Wang S. CXCR4 and anti-BCMA CAR co-modified natural killer cells suppress multiple myeloma progression in a xenograft mouse model. Cancer Gene Ther. (2022) 29:475–83. doi: 10.1038/s41417-021-00365-x 34471234

[17] Kremer KN Dinkel BA Sterner RM Osborne DG Jevremovic D Hedin KE. TCR-CXCR4 signaling stabilizes cytokine mRNA transcripts via a PREX1-Rac1 pathway: implications for CTCL. Blood. (2017) 130:982–94. doi: 10.1182/blood-2017-03-770982 PMC5570680 28694325

[18] Ng YY Tay JCK Wang S. CXCR1 expression to improve anti-cancer efficacy of intravenously injected CAR-NK cells in mice with peritoneal xenografts. Mol Ther Oncolytics. (2020) 16:75–85. doi: 10.1016/j.omto.2019.12.006 31970285 PMC6965500

[19] Muller N Michen S Tietze S Topfer K Schulte A Lamszus K. Engineering NK cells modified with an EGFRvIII-specific chimeric antigen receptor to overexpress CXCR4 improves immunotherapy of CXCL12/SDF-1alpha-secreting glioblastoma. J Immunother. (2015) 38:197–210. doi: 10.1097/CJI.0000000000000082 25962108 PMC4428685

[20] Kremer V Ligtenberg MA Zendehdel R Seitz C Duivenvoorden A Wennerberg E. Genetic engineering of human NK cells to express CXCR2 improves migration to renal cell carcinoma. J Immunother Cancer. (2017) 5:73. doi: 10.1186/s40425-017-0275-9 28923105 PMC5604543

[21] Lee J Kang TH Yoo W Choi H Jo S Kong K. An antibody designed to improve adoptive NK-cell therapy inhibits pancreatic cancer progression in a murine model. Cancer Immunol Res. (2019) 7:219–29. doi: 10.1158/2326-6066.CIR-18-0317 30514792

[22] Ponzetta A Benigni G Antonangeli F Sciume G Sanseviero E Zingoni A. Multiple myeloma impairs bone marrow localization of effector natural killer cells by altering the chemokine microenvironment. Cancer Res. (2015) 75:4766–77. doi: 10.1158/0008-5472.CAN-15-1320 26438594

[23] Bonanni V Antonangeli F Santoni A Bernardini G. Targeting of CXCR3 improves anti-myeloma efficacy of adoptively transferred activated natural killer cells. J Immunother Cancer. (2019) 7:290. doi: 10.1186/s40425-019-0751-5 31699153 PMC6839099

[24] Gunesch JT Dixon AL Ebrahim TA Berrien-Elliott MM Tatineni S Kumar T. CD56 regulates human NK cell cytotoxicity through Pyk2. Elife. (2020) 9:1–28. doi: 10.7554/eLife.57346 PMC7358009 32510326

[25] Gwalani LA Orange JS. Single degranulations in NK cells can mediate target cell killing. J Immunol. (2018) 200:3231–43. doi: 10.4049/jimmunol.1701500 PMC6020067 29592963

[26] Prager I Liesche C van Ooijen H Urlaub D Verron Q Sandstrom N. NK cells switch from granzyme B to death receptor-mediated cytotoxicity during serial killing. J Exp Med. (2019) 216:2113–27. doi: 10.1084/jem.20181454 PMC6719417 31270246

[27] Spear P Wu MR Sentman ML Sentman CL. NKG2D ligands as therapeutic targets. Cancer Immun. (2013) 13:8. 23833565 PMC3700746

[28] Upshaw JL Schoon RA Dick CJ Billadeau DD Leibson PJ. The isoforms of phospholipase C-gamma are differentially used by distinct human NK activating receptors. J Immunol. (2005) 175:213–8. doi: 10.4049/jimmunol.175.1.213 15972651

[29] Abel AM Yang C Thakar MS Malarkannan S. Natural killer cells: development, maturation, and clinical utilization. Front Immunol. (2018) 9:1869. doi: 10.3389/fimmu.2018.01869 30150991 PMC6099181

[30] Vrazo AC Hontz AE Figueira SK Butler BL Ferrell JM Binkowski BF. Live cell evaluation of granzyme delivery and death receptor signaling in tumor cells targeted by human natural killer cells. Blood. (2015) 126:e1–e10. doi: 10.1182/blood-2015-03-632273 26124495 PMC4543232

[31] Bernardini G Sciume G Santoni A. Differential chemotactic receptor requirements for NK cell subset trafficking into bone marrow. Front Immunol. (2013) 4:12. doi: 10.3389/fimmu.2013.00012 23386850 PMC3558687

[32] Biondi M Tettamanti S Galimberti S Cerina B Tomasoni C Piazza R. Selective homing of CAR-CIK cells to the bone marrow niche enhances control of the Acute Myeloid Leukemia burden. Blood. (2023) 141:2587–98. doi: 10.1182/blood.2022018330 PMC10646802 36787509

[33] Laufer JM Kindinger I Artinger M Pauli A Legler DF. CCR7 is recruited to the immunological synapse, acts as co-stimulatory molecule and drives LFA-1 clustering for efficient T cell adhesion through ZAP70. Front Immunol. (2018) 9:3115. doi: 10.3389/fimmu.2018.03115 30692994 PMC6339918

[34] Gollmer K Asperti-Boursin F Tanaka Y Okkenhaug K Vanhaesebroeck B Peterson JR. CCL21 mediates CD4+ T-cell costimulation via a DOCK2/Rac-dependent pathway. Blood. (2009) 114:580–8. doi: 10.1182/blood-2009-01-200923 PMC2713469 19451552

[35] Molon B Gri G Bettella M Gomez-Mouton C Lanzavecchia A Martinez AC. T cell costimulation by chemokine receptors. Nat Immunol. (2005) 6:465–71. doi: 10.1038/ni1191 15821738

[36] Bluhm J Kieback E Marino SF Oden F Westermann J Chmielewski M. CAR T cells with enhanced sensitivity to B cell maturation antigen for the targeting of B cell Non-Hodgkin’s lymphoma and multiple myeloma. Mol Ther. (2018) 26:1906–20. doi: 10.1016/j.ymthe.2018.06.012 PMC6094395 30078440

[37] Kawai T Malech HL. WHIM syndrome: congenital immune deficiency disease. Curr Opin Hematol. (2009) 16:20–6. doi: 10.1097/MOH.0b013e32831ac557 PMC2673024 19057201

[38] Hernandez PA Gorlin RJ Lukens JN Taniuchi S Bohinjec J Francois F. Mutations in the chemokine receptor gene CXCR4 are associated with WHIM syndrome, a combined immunodeficiency disease. Nat Genet. (2003) 34:70–4. doi: 10.1038/ng1149 12692554

[39] Balabanian K Lagane B Pablos JL Laurent L Planchenault T Verola O. WHIM syndromes with different genetic anomalies are accounted for by impaired CXCR4 desensitization to CXCL12. Blood. (2005) 105:2449–57. doi: 10.1182/blood-2004-06-2289 15536153

[40] Kawai T Choi U Whiting-Theobald NL Linton GF Brenner S Sechler JM. Enhanced function with decreased internalization of carboxy-terminus truncated CXCR4 responsible for WHIM syndrome. Exp Hematol. (2005) 33:460–8. doi: 10.1016/j.exphem.2005.01.001 15781337

[41] Levy E Reger R Segerberg F Lambert M Leijonhufvud C Baumer Y. Enhanced bone marrow homing of natural killer cells following mRNA transfection with gain-of-function variant CXCR4(R334X). Front Immunol. (2019) 10:1262. doi: 10.3389/fimmu.2019.01262 31231387 PMC6560173

[42] McDermott DH Lopez J Deng F Liu Q Ojode T Chen H. AMD3100 is a potent antagonist at CXCR4(R334X), a hyperfunctional mutant chemokine receptor and cause of WHIM syndrome. J Cell Mol Med. (2011) 15:2071–81. doi: 10.1111/j.1582-4934.2010.01210.x PMC3071896 21070597

[43] Kim HY Moon JY Ryu H Choi YS Song IC Lee HJ. Bortezomib inhibits the survival and proliferation of bone marrow stromal cells. Blood Res. (2015) 50:87–96. doi: 10.5045/br.2015.50.2.87 26157778 PMC4486164

[44] Gunesch JT Angelo LS Mahapatra S Deering RP Kowalko JE Sleiman P. Genome-wide analyses and functional profiling of human NK cell lines. Mol Immunol. (2019) 115:64–75. doi: 10.1016/j.molimm.2018.07.015 30054012 PMC6345623

[45] Alter G Malenfant JM Altfeld M. CD107a as a functional marker for the identification of natural killer cell activity. J Immunol Methods. (2004) 294:15–22. doi: 10.1016/j.jim.2004.08.008 15604012

[46] Lorenzo-Herrero S Sordo-Bahamonde C Gonzalez S Lopez-Soto A. CD107a degranulation assay to evaluate immune cell antitumor activity. Methods Mol Biol. (2019) 1884:119–30. doi: 10.1007/978-1-4939-8885-3_7 30465198

[47] Peacock JW Jirik FR. TCR activation inhibits chemotaxis toward stromal cell-derived factor-1: evidence for reciprocal regulation between CXCR4 and the TCR. J Immunol. (1999) 162:215–23. doi: 10.4049/jimmunol.162.1.215 9886389

[48] Dar WA Knechtle SJ. CXCR3-mediated T-cell chemotaxis involves ZAP-70 and is regulated by signalling through the T-cell receptor. Immunology. (2007) 120:467–85. doi: 10.1111/j.1365-2567.2006.02534.x PMC2265907 17250586

[49] Felce JH Parolini L Sezgin E Cespedes PF Korobchevskaya K Jones M. Single-molecule, super-resolution, and functional analysis of G protein-coupled receptor behavior within the T cell immunological synapse. Front Cell Dev Biol. (2020) 8:608484. doi: 10.3389/fcell.2020.608484 33537301 PMC7848080

[50] Bromley SK Peterson DA Gunn MD Dustin ML. Cutting edge: hierarchy of chemokine receptor and TCR signals regulating T cell migration and proliferation. J Immunol. (2000) 165:15–9. doi: 10.4049/jimmunol.165.1.15 10861029

[51] Ahr B Denizot M Robert-Hebmann V Brelot A Biard-Piechaczyk M. Identification of the cytoplasmic domains of CXCR4 involved in Jak2 and STAT3 phosphorylation. J Biol Chem. (2005) 280:6692–700. doi: 10.1074/jbc.M408481200 15615703

[52] Li Y Basar R Wang G Liu E Moyes JS Li L. KIR-based inhibitory CARs overcome CAR-NK cell trogocytosis-mediated fratricide and tumor escape. Nat Med. (2022) 28:2133–44. doi: 10.1038/s41591-022-02003-x PMC9942695 36175679

[53] Davenport AJ Cross RS Watson KA Liao Y Shi W Prince HM. Chimeric antigen receptor T cells form nonclassical and potent immune synapses driving rapid cytotoxicity. Proc Natl Acad Sci U S A. (2018) 115:E2068–E76. doi: 10.1073/pnas.1716266115 PMC5834689 29440406

[54] Teppert K Wang X Anders K Evaristo C Lock D Kunkele A. Joining forces for cancer treatment: from “TCR versus CAR” to “TCR and CAR. Int J Mol Sci. (2022) 23. doi: 10.3390/ijms232314563 PMC9739809 36498890

[55] Li R Ma C Cai H Chen W. The CAR T-cell mechanoimmunology at a glance. Adv Sci (Weinh). (2020) 7:2002628. doi: 10.1002/advs.202002628 33344135 PMC7740088

[56] Gudipati V Rydzek J Doel-Perez I Goncalves VDR Scharf L Konigsberger S. Inefficient CAR-proximal signaling blunts antigen sensitivity. Nat Immunol. (2020) 21:848–56. doi: 10.1038/s41590-020-0719-0 32632291

[57] Kremer KN Humphreys TD Kumar A Qian NX Hedin KE. Distinct role of ZAP-70 and Src homology 2 domain-containing leukocyte protein of 76 kDa in the prolonged activation of extracellular signal-regulated protein kinase by the stromal cell-derived factor-1 alpha/CXCL12 chemokine. J Immunol. (2003) 171:360–7. doi: 10.4049/jimmunol.171.1.360 12817019

[58] Majzner RG Rietberg SP Sotillo E Dong R Vachharajani VT Labanieh L. Tuning the antigen density requirement for CAR T-cell activity. Cancer Discov. (2020) 10:702–23. doi: 10.1158/2159-8290.CD-19-0945 PMC7939454 32193224

[59] Contento RL Molon B Boularan C Pozzan T Manes S Marullo S. CXCR4-CCR5: a couple modulating T cell functions. Proc Natl Acad Sci USA. (2008) 105:10101–6. doi: 10.1073/pnas.0804286105 PMC2481367 18632580

[60] Kumar A Humphreys TD Kremer KN Bramati PS Bradfield L Edgar CE. CXCR4 physically associates with the T cell receptor to signal in T cells. Immunity. (2006) 25:213–24. doi: 10.1016/j.immuni.2006.06.015 16919488

[61] Dinkel BA Kremer KN Rollins MR Medlyn MJ Hedin KE. GRK2 mediates TCR-induced transactivation of CXCR4 and TCR-CXCR4 complex formation that drives PI3Kgamma/PREX1 signaling and T cell cytokine secretion. J Biol Chem. (2018) 293:14022–39. doi: 10.1074/jbc.RA118.003097 PMC6130939 30018141

[62] Nakayama T Hieshima K Nagakubo D Sato E Nakayama M Kawa K. Selective induction of Th2-attracting chemokines CCL17 and CCL22 in human B cells by latent membrane protein 1 of Epstein-Barr virus. J Virol. (2004) 78:1665–74. doi: 10.1128/JVI.78.4.1665-1674.2004 PMC369498 14747532

[63] Chockley PJ Ibanez-Vega J Krenciute G Talbot LJ Gottschalk S. Synapse-tuned CARs enhance immune cell anti-tumor activity. Nat Biotechnol. (2023) 41:1434–45. doi: 10.1038/s41587-022-01650-2 PMC10394118 36732477

[64] Maude SL Laetsch TW Buechner J Rives S Boyer M Bittencourt H. Tisagenlecleucel in children and young adults with B-cell lymphoblastic leukemia. N Engl J Med. (2018) 378:439–48. doi: 10.1056/NEJMoa1709866 PMC5996391 29385370

[65] Fry TJ Shah NN Orentas RJ Stetler-Stevenson M Yuan CM Ramakrishna S. CD22-targeted CAR T cells induce remission in B-ALL that is naive or resistant to CD19-targeted CAR immunotherapy. Nat Med. (2018) 24:20–8. doi: 10.1038/nm.4441 PMC5774642 29155426

[66] Turtle CJ Hanafi LA Berger C Gooley TA Cherian S Hudecek M. CD19 CAR-T cells of defined CD4+:CD8+ composition in adult B cell ALL patients. J Clin Invest. (2016) 126:2123–38. doi: 10.1172/JCI85309 PMC4887159 27111235

[67] Bunnell SC Hong DI Kardon JR Yamazaki T McGlade CJ Barr VA. T cell receptor ligation induces the formation of dynamically regulated signaling assemblies. J Cell Biol. (2002) 158:1263–75. doi: 10.1083/jcb.200203043 PMC2173229 12356870

[68] Katz ZB Novotna L Blount A Lillemeier BF. A cycle of Zap70 kinase activation and release from the TCR amplifies and disperses antigenic stimuli. Nat Immunol. (2017) 18:86–95. doi: 10.1038/ni.3631 27869819 PMC5490839

[69] Naramura M Jang IK Kole H Huang F Haines D Gu H. c-Cbl and Cbl-b regulate T cell responsiveness by promoting ligand-induced TCR down-modulation. Nat Immunol. (2002) 3:1192–9. doi: 10.1038/ni855 12415267

[70] Yang CW Hojer CD Zhou M Wu X Wuster A Lee WP. Regulation of T cell receptor signaling by DENND1B in TH2 cells and allergic disease. Cell. (2016) 164:141–55. doi: 10.1016/j.cell.2015.11.052 26774822

[71] Davenport AJ Jenkins MR Cross RS Yong CS Prince HM Ritchie DS. CAR-T cells inflict sequential killing of multiple tumor target cells. Cancer Immunol Res. (2015) 3:483–94. doi: 10.1158/2326-6066.CIR-15-0048 25711536

[72] Walker AJ Majzner RG Zhang L Wanhainen K Long AH Nguyen SM. Tumor antigen and receptor densities regulate efficacy of a chimeric antigen receptor targeting anaplastic lymphoma kinase. Mol Ther. (2017) 25:2189–201. doi: 10.1016/j.ymthe.2017.06.008 PMC5589087 28676342

[73] Li W Qiu S Chen J Jiang S Chen W Jiang J. Chimeric antigen receptor designed to prevent ubiquitination and downregulation showed durable antitumor efficacy. Immunity. (2020) 53:456–70 e6. doi: 10.1016/j.immuni.2020.07.011 32758419

