# Supplementary material for: CXCR4 has a dual role in improving the efficacy of BCMA-redirected CAR-NK cells in multiple myeloma
Source: Front Immunol. 2024 Jun 24;15:1383136. doi: 10.3389/fimmu.2024.1383136 (PMC11228140; doi:10.3389/fimmu.2024.1383136)
Supplement: Supplementary file 1 [file DataSheet_1.pdf]

Supplementary Material

Supplemental methods

Cell lines

The NK cell line NK-92 (CRL-2408) was obtained from ATCC and was grown in α-MEM medium supplemented with 12.5% horse serum, 12.5% FBS, 0.2mM inositol, 0.1mM 2-mercaptoethanol, 0.02 mM folate, 100 U/ml recombinant IL-2 (Peprotech), 2 mM L-glutamine, 100 U/ml penicillin, 0,1 mg/ml streptomycin, 1% sodium pyruvate (all from Gibco).

The NK cell line YTS was maintained in RPMI-1640 with 10% FBS, 2 mM L-glutamine, 10 mM Hepes, 100 U/ml penicillin, 0,1 mg/ml streptomycin. Cell lines were cultivated at 37°C in a humidified incubator. Upon receipt of the NK cell lines, they were immediately expanded and aliquots were frozen at early passage numbers. Further confirmation of cell identity was done by flow cytometry (CD56+CD3-CD19-). The Burkitt lymphoma B cell line RAJI (ACC319) was purchased from DSMZ (Braunschweig). All cultures were tested Mycoplasma-free prior to usage.

Production of retroviral supernatant

Production of viral supernatants was done essentially as described (36). Briefly, amphotropic retroviral supernatants were produced by stable packaging cell lines based on 293Vec-Galv. Transient transfection of HEK293T cells was performed using the appropriate MP71 construct and two plasmids encoding the MLV *env* (pALF-10A1GaV) and *gag*/*pol* (pcDNA3.1-MLV gag/pol) genes in a 1:1:1 ratio. Retroviral supernatants were collected 48 hrs post-transfection, filtered and either used directly for transduction or stored at -80°C.

Retroviral transduction of primary human NK cells, NK cell lines and reporter cells

Human PBMCs were isolated from peripheral blood of healthy voluntary donors, followed by density gradient centrifugation. Alternatively, leukapheresis material from anonymous healthy blood donors was used. The recruitment of voluntary blood donors was conducted according to the declaration of Helsinki and in accordance with local ethical guidelines. Enrichment of CD56+ NK cells was achieved by magnetic separation, employing the "NK Cell Isolation Kit, human" (Miltenyi Biotec). NK cells were stimulated with "NK Cell Activation/Expansion Kit" beads (Miltenyi Biotec), and further cultured in NK MACS medium (Miltenyi Biotec) supplemented with 5% human AB serum (Sigma-Aldrich) and 500 U/ml IL-2, 20 ng/ml IL-15 (Peprotech).

For γ-retroviral transduction at day 7 of the NK cell culture, a 24-well non tissue treated cell culture plate was incubated with 250 µl recombinant human fibronectin fragment solution (RetroNectin®, Takara) (12.5 µg/ml) per well for 2h at 37°C or overnight at 4°C. The RetroNectin solution was removed and the plate blocked with 500 µl per well 2% BSA in PBS for 30 min at 37°C. Plates were washed with 2 ml per well 25 mM HEPES in PBS, and 500 µl virus supernatant per well was added. The plates were then centrifuged at 3000 g at 4°C for 90 minutes. The supernatant was removed and wells were filled with $1x10^6$ cells, 250 µL of fresh virus supernatant and 750 µL cell culture medium, including IL-2 and IL-15. Polybrene was added to a final concentration of 5 µg/mL. For spinoculation, plates were centrifuged at 800 g, 32°C for 90 minutes. Fresh complete medium supplemented with IL-2 and IL-15 was added

every 2-3 days after transduction. Transgene expression was confirmed 5 days after transduction. Sorted cells were further expanded for at least 5 days before migration assays were performed.
Transduced NK-92 and YTS cells were cultured and FACS-sorted similarly to the primary NK cells. $BCMA^+$ Raji target cells were generated by stable transduction with BCMA. A γ-retrovirus was produced in 293Vec-Galv cells.

Flow cytometry
Prior to antibody staining, NK cells were blocked with Human TruStain FcX Fc-receptor blockage solution for 15 min (Biolegend). To discriminate between living and dead cells, stained samples were incubated with 7-AAD (BioLegend) 5 to 10 min before data acquisition or stained with LIVE/DEAD™ Fixable Aqua Dead Cell Stain Kit (Molecular Probes) prior to antibody staining.
The following fluorochrome-conjugated antibodies (all from BioLegend) against NK cell or and target cell antigens were used for analysis by flow cytometry:
CD56 (Alexa Fluor 647, cl. MEM-188), CD16 (FITC, PE; cl. 3G8), CD3 (PE, FITC, Brilliant Violet421, cl. UCHT1), CD19 (FITC, cl. HIB19), CXCR4 (Brilliant Violet421, cl. 12G5), BCMA (Phycoerythrin (PE), cl. 19F2), CD107a (PE, cl. H4A3), CD11b (APC, cl. ICRF44), CD11a/CD18 (PE, LFA-1, cl. M24), CD44 (FITC, cl. IM7), CD62L (Pacific Blue, cl. DREG-56), CD38 (FITC, cl. HIT2), CD138 (APC, cl. DL-101), BCMA (CD269) (PE, cl. 19F2). Analytical samples were acquired on either a FACS Canto II, Symphony A1 flow cytometer (BD Biosciences), or a MACSQuant 10 analyzer (Miltenyi Biotec). The data were analyzed with FlowJo v. 10.0.8 software (Tree Star).
Primary NK cells and NK cell lines were FACS sorted using a FITC-coupled BCMA-peptide (aa 1-54; ACRO Biosystems). All cell-sorting steps were carried out on a “FACS Aria III” or “FACS Aria Fusion” instrument (BD Biosciences).

Migration assay
A transwell migration assay to determine the migratory capacity of CAR-transduced NK cells was done essentially as described (Heinig et al., 2014; Hopken et al., 2002). Briefly, assays for NK-92 cells were performed in 5 µm-pore Transwell plates (Corning) for 4 hours at 37°C, coated with collagen IV. CXCL12 (R&D Research Diagnostics) was used at a concentration of 25 or 100 ng/ml, respectively. For primary NK cells, collagen I and 3 µm pore Transwell plates were used. Migration medium contained α-MEM, 2-mercaptoethanol, inositol, Pen/Strep, and 1% BSA.
Cells in the lower chamber were counted manually, and the migration factor was calculated relative to the number of cells migrated without CXCL12.

Degranulation assay
CAR-transduced NK-92 cells were co-cultured with target cells in a 96-well U-bottom plate in a 1:1 ratio ($5x10^4$ cells per well each) for 30 min or 90 min at 37°C. Anti-human CD107a-PE antibody (H4A3, Becton Dickinson (BD) Biosciences) was added directly to the co-culture. At the end of the stimulation period, cells were washed once in ice cold FACS buffer. Cells were blocked for 15 min by addition of Human TruStain FcX (block FcR binding; BioLegend), followed by staining with anti-human CD56-AF647 (cl. MEM-188, BioLegend) and anti-human CD19-FITC (HIB19, BioLegend) antibodies to distinguish effector cells and the $Raji^{BCMA}$ target

cells. Cells were washed, and 7-AAD was added for dead cell detection. Degranulation rate was calculated as percent of CD107A-positive $CD56^{+}CD19^{-}$ NK-92 cells.

Immunological synapse formation

An equal number of $1x10^{5}$ CAR-transduced NK-92 cells and $Raji^{BCMA}$ target cells were mixed in a total volume of 200 μl pre-warmed α-MEM medium in an Eppendorf tube. Cells were allowed to form synapses for 5 min at 37°C in a waterbath, and the process was stopped by direct adding a 4% PFA-containing Histofix solution to the cell suspension. Cells were pipetted gently and transferred into cytofunnels of a cytospin centrifuge (Shandon). Cells were transferred onto glass slides by spinning for 3 min at 1.200 rpm. Slides were then further fixed with Histofix for 10 min at RT, washed in TBS and prior to antibody staining, blocked with TrueStain blocking reagent in permeabilization buffer containing 0.1% Triton-X100 for 15 min.

Cell conjugates were stained with antibodies directed against phospho-ZAP70 (rabbit, P-ZAP70 Y319/Y352 (Syk); Cell Signaling), BCMA-APC, CXCR4-BV421, and with Phalloidin-AF594 conjugate (Sigma-Aldrich) for actin network visualization. For detection of phospho-ZAP-70 primary antibody, a goat-anti-rabbit Ig AF568 was used (Southern Biotech). Slides were washed in TBST-Tween, and mounted in Dako Immumount.

Images for quantification were acquired on a Zeiss Axio Imager M2m widefield microscope. Images were obtained with a 40x Plan-Apochromat NA 0.95 objective. Digital images were processed with Axio Vision 4.8.2 software (Carl Zeiss). For quantitative image analysis, ImageJ 2.0.0 software was used, and images were saved in TIFF format.

Exemplary images were also acquired on an LSM-980 airyscan confocal microscope, equipped with a 63x Plan Apochromat NA 1.40 oil objective. Digital images were obtained with ZEN software (Zeiss), and further processed with Fiji.

Immunoblotting

Lysates of NK-92 cells were generated in RIPA buffer supplemented with phenylmethysulfonyl fluoride (PMSF, 1 mM) and aprotinin (5 μg/ml), in addition, phosphatase inhibitor tablets (Roche, Sigma) were used. Lysates were separated by denaturing SDS-PAGE and transfer onto a nitrocellulose membrane (GE Healthcare). Membranes were blocked with 0.5% BSA or 5 % milk in TBST and washed with TBST. Primary antibodies were visualized by fluorochrome-coupled or HRP-linked secondary antibodies. Blots were imaged using a ChemiDoc MP imaging system (BioRead). Densitometric quantification of gel bands was performed using ImageJ 1.440 software.

Antibodies for immunoblot analysis

The following primary antibodies were used for immunoblot analysis: anti-phospho-pZAP-70(Tyr319/pSyk(Tyr352), anti-phospho-p44/42 MAPK (ERK1/2) (Thr202/Tyr204), anti-p44/42 MAPK(ERK1/2), anti-phospho-AKT, anti-AKT (Pan)(C67E7) (all from Cell Signaling), anti-Calnexin (Enzo Life Science) was used as loading control.

Supplemental figure legends

Figure S1. The NK-92 cell line lacks CXCR4 expression
(A, B) Flow cytometry analysis of chemokine receptor expression on NK-92 cells. Histograms depict CXCR4 expression, compared to CXCR3 and CCR6 expression. Grey filled graph, specific antibody versus open graph, isotype control.
C) Peripheral NK cells were isolated from peripheral blood and enriched by MACS bead sorting. NK cells were stimulated with "NK Cell Activation/Expansion Kit" beads and cultured in medium supplemented with 5% human AB serum and 500 U/ml IL-2, 20 ng/ml IL-15. At day 7, NK cells were retrovirally transduced with the CAR and CAR-CXCR4 constructs. At day 9 transduced NK-92 cells were analyzed by FACS for CAR and CXCR4 expression.
D) Quantification of P-ERK1,2$^{T202/Y204}$ occurrence; this quantification refers to **Fig. 1H**. The ratio of total ERK1,2 and phosphorylated P-ERK1,2 is given (n= 3 independent experiments). Error bars ± SEM.
E) NK-92 cells stably transduced with CAR and CAR-CXCR4 were stimulated for 5 min with 25 ng/ml CXCL12. Cell lysates were probed for phosphorylated AKT, total AKT, and Calnexin as a further loading control. Representative immunoblots are given.
F) Quantification of CXCL12-induced AKT phosphorylation, showing CAR and CAR-CXCR4 transduced NK-92 cells. The ratio of total AKT and phosphorylated AKT is given. Error bars ± SEM. In **D)** and **F)**, data points represent independent biological replicates.

Figure S2. Workflow for reporter cell line generation
Raji cells were transduced with a γ-retrovirus encoding BCMA. At day 6 after transduction, cells were enriched by FACS sorting for homogenous BCMA expression, further expanded and several aliquots were frozen for later experiments where the cell line served as a target cell for CAR NK cell stimulation.

Figure S3. CXCR4 expression endows the NK cell line YTS with enhanced cytolytic efficacy
A) FACS plots and histograms depicting the generation of CAR^{+} YTS cells. YTS cells underwent retroviral transduction with the CAR and CAR-CXCR4 constructs. BCMA-CAR^{+} expressing YTS cells, as detected by cognate FITC-conjugated BCMA-peptide, were enriched twice by FACS to generate stable CAR and CAR-CXCR4 YTS cell lines. Isotype control, grey filled histogram.
B) Exactly as in **Fig. 2A, B)**, a flow cytometry-based cytotoxicity assay was performed by co-culturing CAR-transduced YTS cells and Raji^{BCMA} target cells. Representative FACS plot depicting a 20-hour co-culture between Raji^{BCMA} cells (CD19^{+}) with either SP6-CAR ctrl., CAR, or CAR-CXCR4 YTS effector cells (CD56^{+}). The percentage of specific-Raji^{BCMA} cell killing is calculated using the formula: %=(1-(tumor cells in coculture/tumor cells only)) x 100.
(dependent upon E:T ratio). Numbers in the gates are the percentages of effector and target cells.
C) Quantification of specific-Raji^{BCMA} cell killing in percent for the aforementioned co-cultures at the indicated E:T ratios (n=4 independent biological replicates). Noteworthy, the cytolytic capacity of YTS cells is reduced compared to NK-92 cells, indicating that B7.1^{low} expressing Raji target cells fail to activate the CD28-dependent co-stimulatory pathway in YTS cell. Statistics calculated by a paired t-test. Error bars ± SEM.

Figure S4. Multiple Myeloma cell-induced CAR NK-92 cell degranulation depends on BCMA expression

A) Representative FACS plot of a degranulation assay detecting LAMP-1 expression on $CD56^+$ SP6-CAR as negative control, CAR, and CAR-CXCR4 NK-92 cells following co-culture with MM.1S-luc.GFP cells for 30-min. Numbers within the gates are the frequencies in percent of LAMP-1^+ cells, numbers in black are without stimulation; in red are the percentages after stimulation.

B) Quantification bar plot of **A**) after 30-min stimulation. Error bars ± SEM; n=3 independent biological replicates.

C) Representative FACS plots of primary bone marrow derived MM specimen. Definition of MM or plasma cells cells by $CD38^{high}$ $CD138^+$ $BCMA^+$ $CD19^-$ expression; this population is highlighted in red color code in middle and right FACS dot plots.

D) Generation of CXCR4 NK-92 cells by retroviral transduction. NK-92 cells after transduction were sorted for CXCR4 overexpression.

E) FACS plot of a degranulation assay for NK-92, CXCR4-NK-92, and CAR-NK92 cells in co-culture with MM.1S-luc.GFP cells for 30 min, as in **A)** and **Fig. 2C.** Numbers on the gates indicate the frequencies of effector cells that degranulated.

F) Quantification bar plots after 30-min stimulation are depicted. Error bars ± SEM; n=3 independent biological replicates. An unpaired t-test was applied.

Figure S5. BCMA-peptide loaded microbeads stimulate IFN-γ release from BCMA-CAR transduced NK-92 cells in a concentration-dependent manner

SP6-CAR, CAR and CAR-CXCR4 exhibiting NK-92 cells were stimulated for 18 hrs with BCMA beads at the concentrations indicated. For maximum release of IFN-γ, NK-92 cells were stimulated with PMA/ionomycin, Min. stands for NK-92 cells without stimulation. After 18 hrs of bead stimulation, cell-free supernatants were harvested, and analyzed by ELISA for IFN-γ content; n=2 independent biological replicates were performed.

Figure S1-S5

Graphical abstract
