# Supplementary figures and images for: CXCR4 has a dual role in improving the efficacy of BCMA-redirected CAR-NK cells in multiple myeloma
Source: Front Immunol. 2024 Jun 24;15:1383136. doi: 10.3389/fimmu.2024.1383136 (PMC11228140; doi:10.3389/fimmu.2024.1383136)

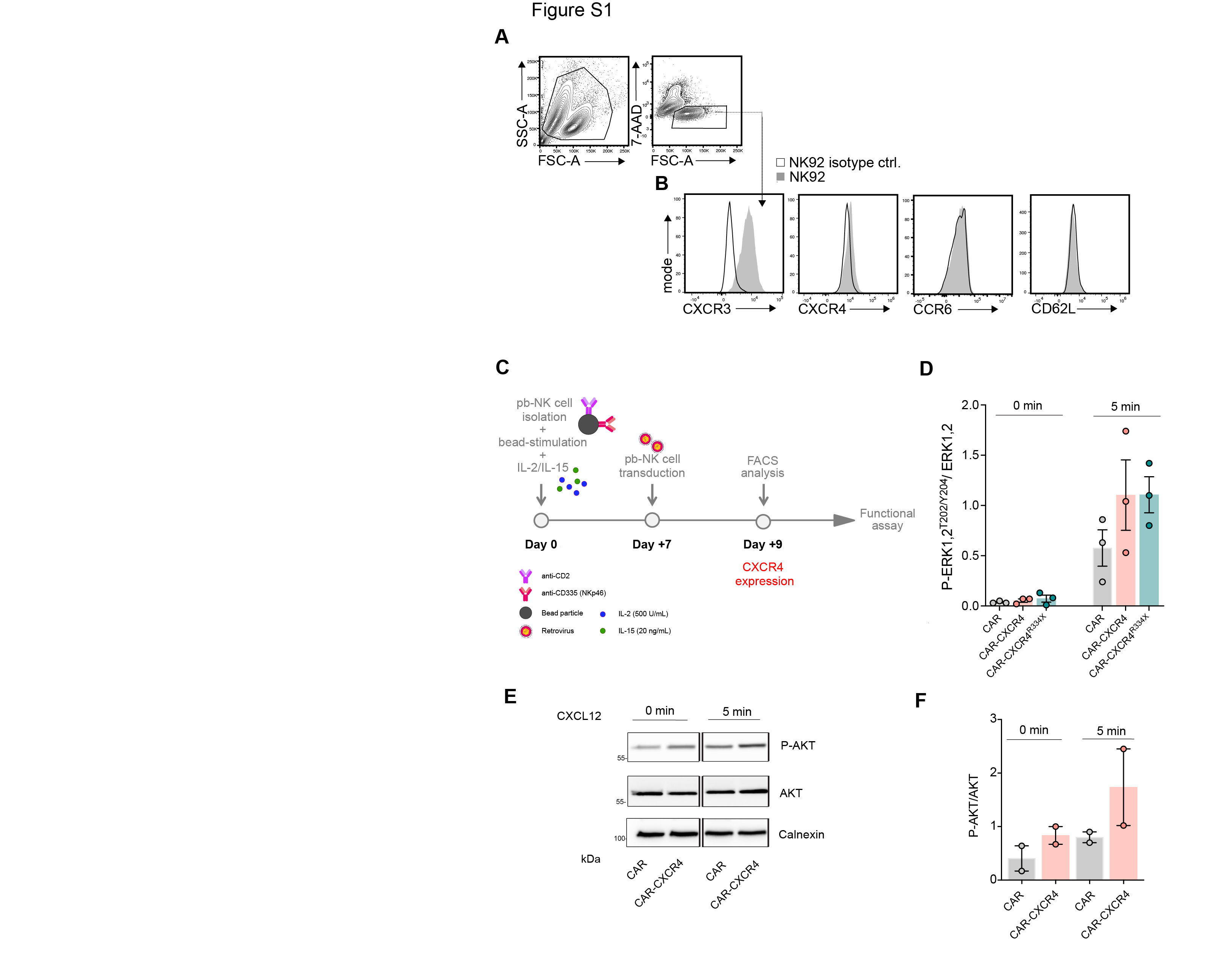

Supplement: Supplementary file 2 [file Image_1.tif]

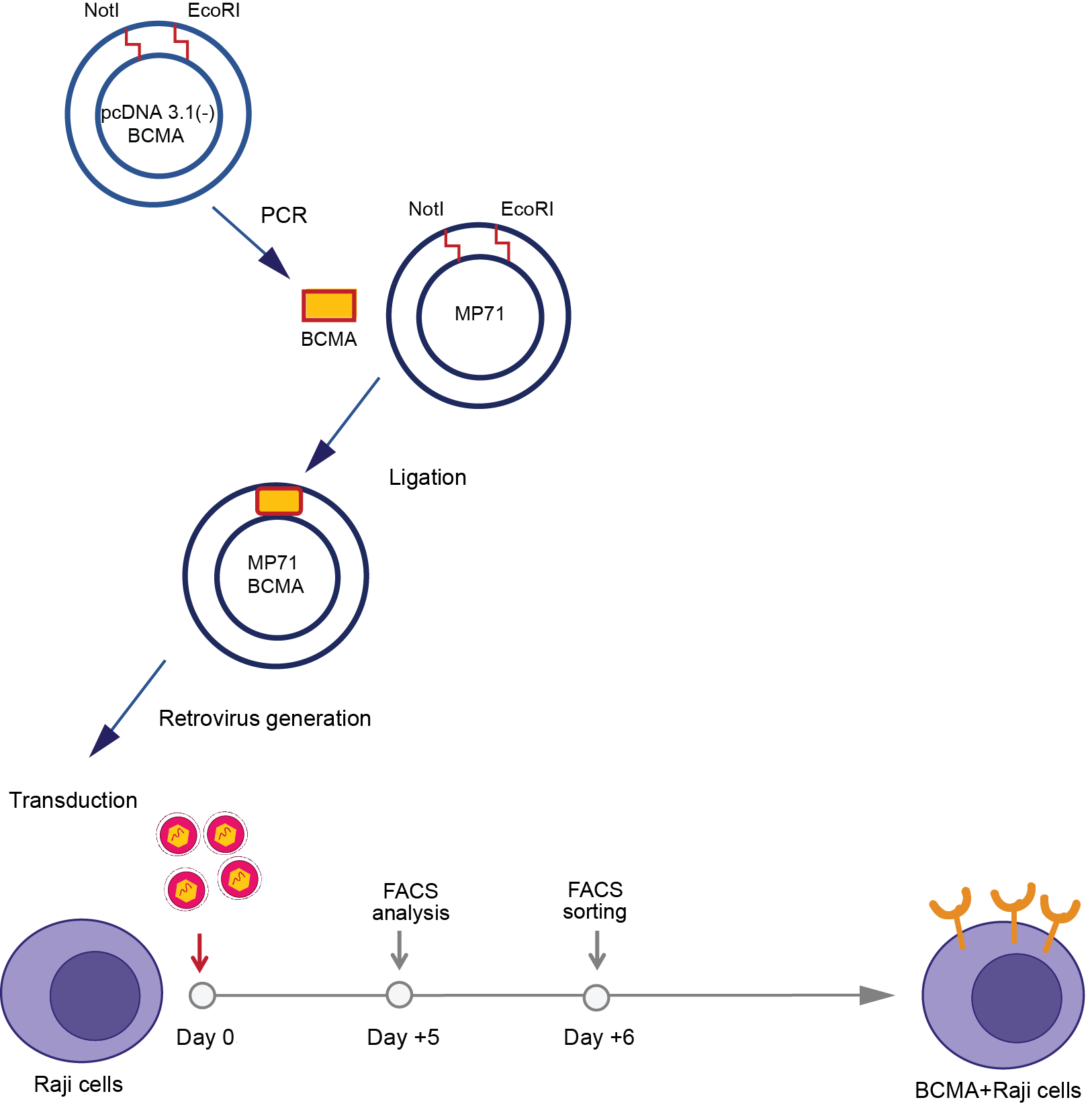

Supplement: Supplementary file 3 [file Image_2.tif]

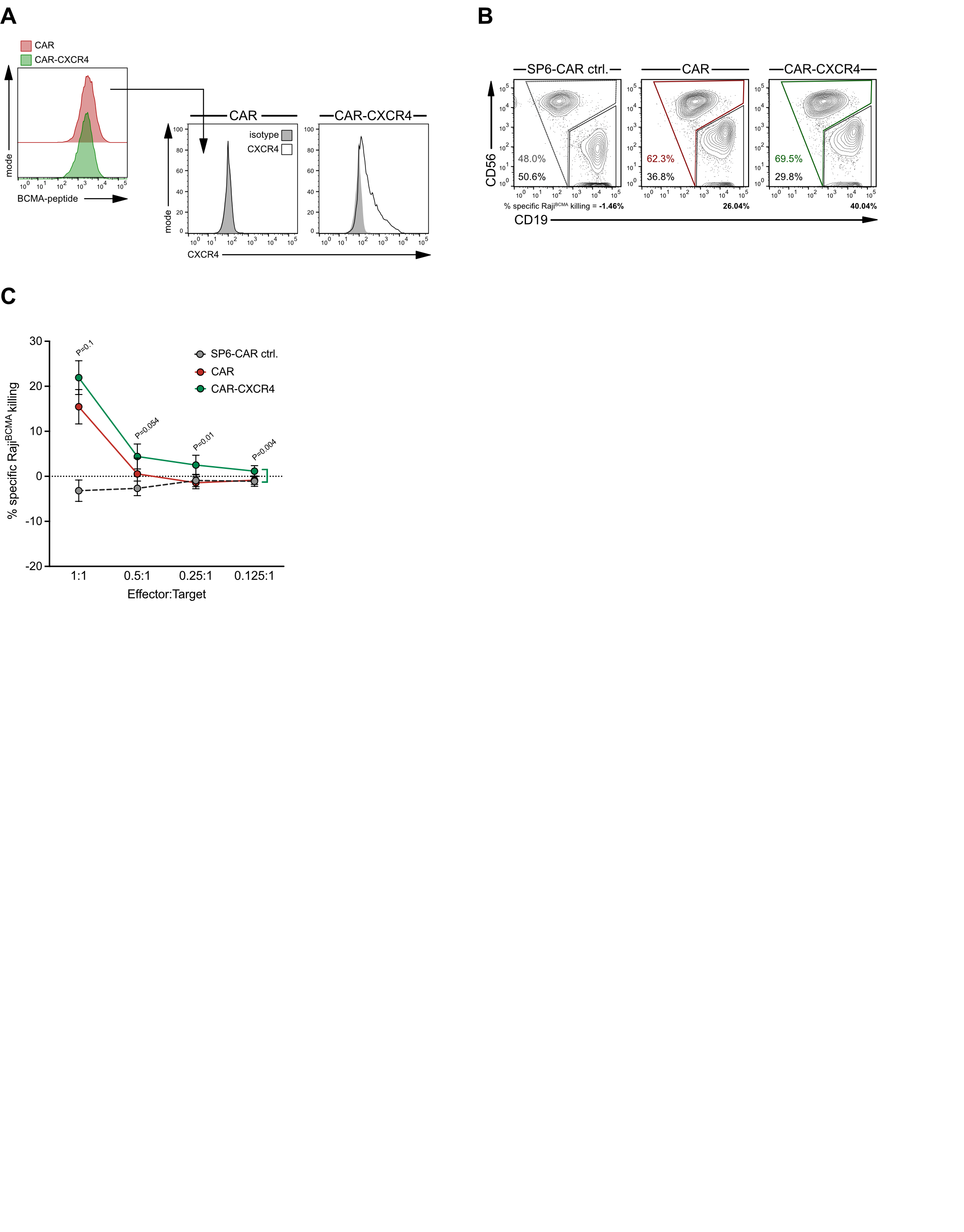

Supplement: Supplementary file 4 [file Image_3.tiff]

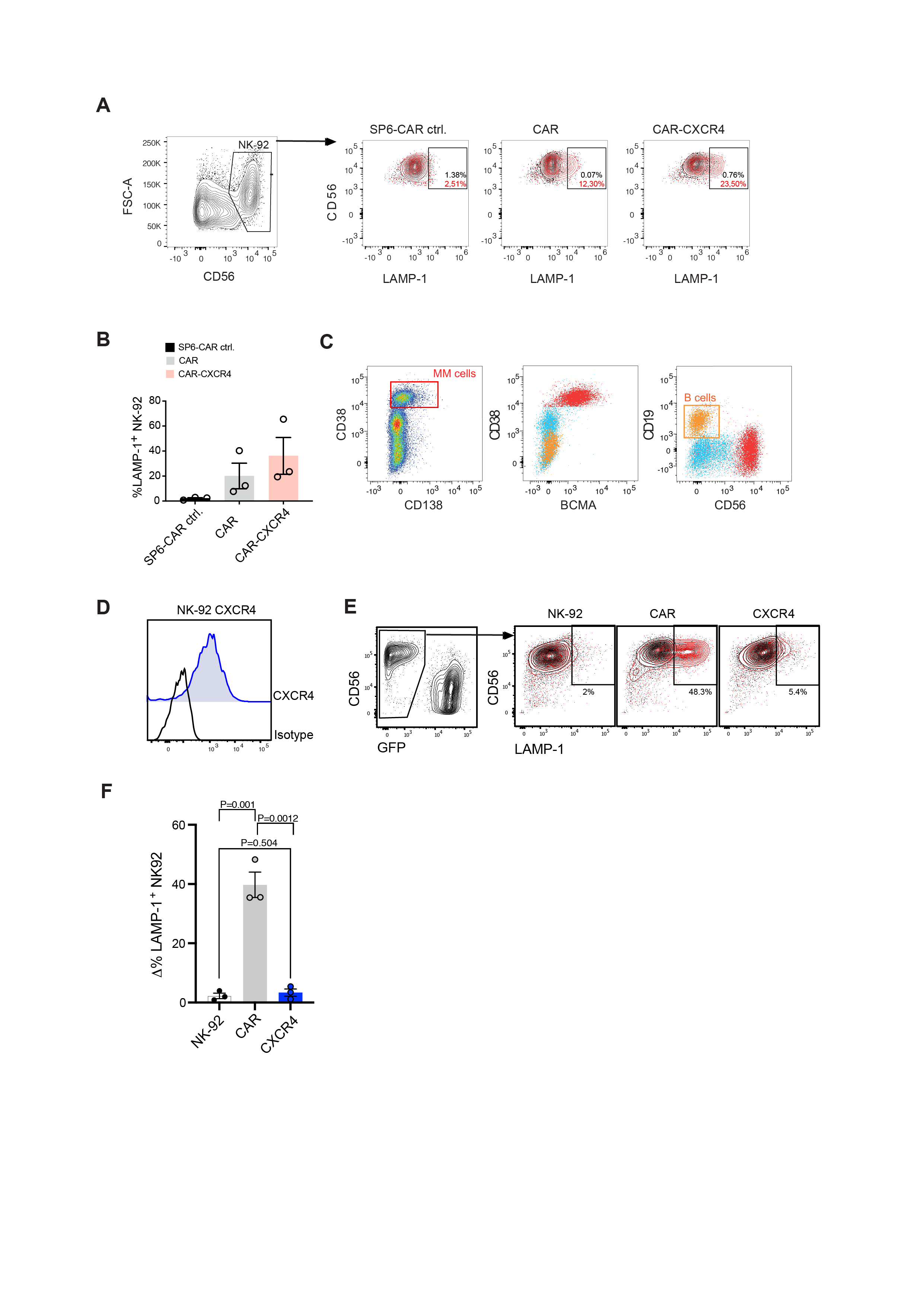

Supplement: Supplementary file 5 [file Image_4.tif]

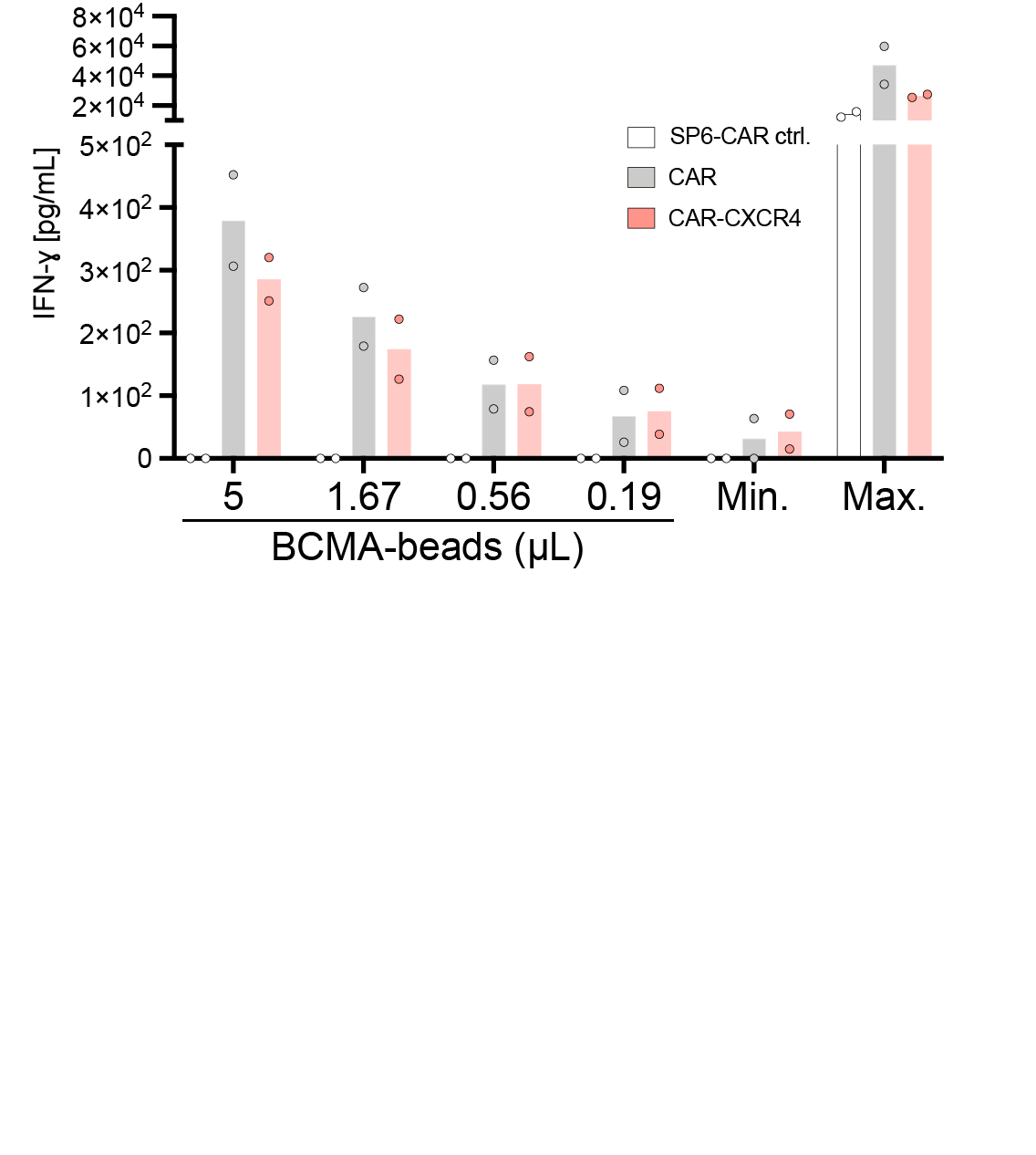

Supplement: Supplementary file 6 [file Image_5.tif]

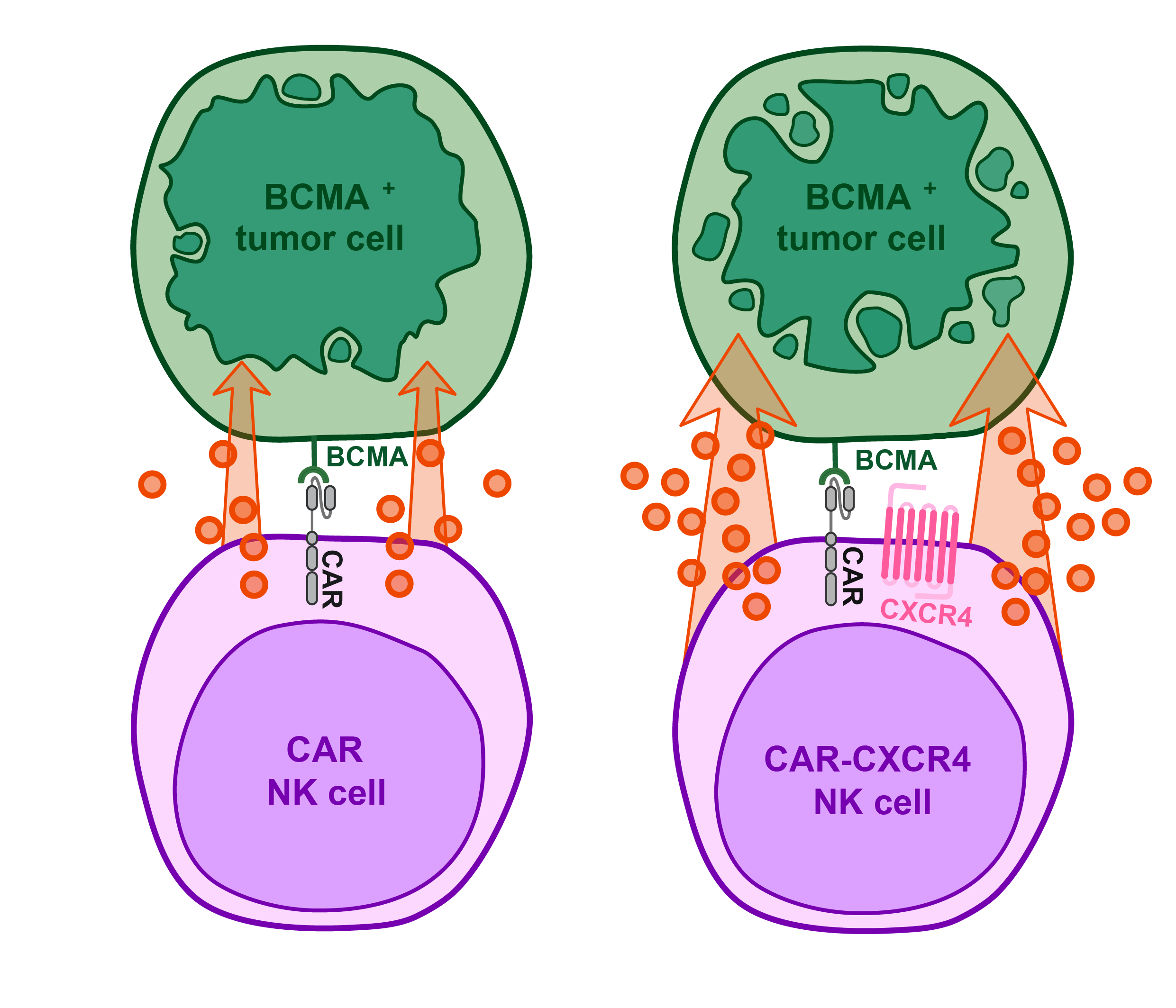

Supplement: Supplementary file 7 [file Image_6.tif]
